# Brain microvessel cross-presentation is a hallmark of experimental cerebral malaria

**DOI:** 10.1002/emmm.201202273

**Published:** 2013-05-16

**Authors:** Shanshan W Howland, Chek Meng Poh, Sin Yee Gun, Carla Claser, Benoit Malleret, Nilabh Shastri, Florent Ginhoux, Gijsbert M Grotenbreg, Laurent Rénia

**Affiliations:** 1 Singapore Immunology Network, Agency for ScienceTechnology and Research (A*STAR), Singapore, Singapore; 2 Department of Microbiology, Life Sciences InstituteYong Loo Lin School of Medicine, National University of Singapore, Singapore, Singapore; 3 Faculty of Science, Department of Biological Sciences, Life Sciences InstituteYong Loo Lin School of Medicine, National University of Singapore, Singapore, Singapore; 4 Immunology Programme, Life Sciences InstituteYong Loo Lin School of Medicine, National University of Singapore, Singapore, Singapore; 5 Division of Immunology and Pathogenesis, Department of Molecular and Cell Biology, University of CaliforniaBerkeley, CA, USA

**Keywords:** brain, cross-presentation, malaria, pathology, T cells

## Abstract

Cerebral malaria is a devastating complication of *Plasmodium falciparum* infection. Its pathogenesis is complex, involving both parasite- and immune-mediated events. CD8^+^ T cells play an effector role in murine experimental cerebral malaria (ECM) induced by *Plasmodium berghei* ANKA (PbA) infection. We have identified a highly immunogenic CD8 epitope in glideosome-associated protein 50 that is conserved across rodent malaria species. Epitope-specific CD8^+^ T cells are induced during PbA infection, migrating to the brain just before neurological signs manifest. They are functional, cytotoxic and can damage the blood–brain barrier *in vivo*. Such CD8^+^ T cells are also found in the brain during infection with parasite strains/species that do not induce neuropathology. We demonstrate here that PbA infection causes brain microvessels to cross-present parasite antigen, while non-ECM-causing parasites do not. Further, treatment with fast-acting anti-malarial drugs before the onset of ECM reduces parasite load and thus antigen presentation in the brain, preventing ECM death. Thus our data suggest that combined therapies targeting both the parasite and host antigen-presenting cells may improve the outcome of CM patients.

→ See accompanying article http://dx.doi.org/10.1002/emmm.201302849

## INTRODUCTION

Malaria remains one of the most important global health problems, affecting more than 200 million people and causing 655,000 deaths in 2010, most of them young children in Africa (World Health Organization, [Bibr b59]). The most severe pathological complication of *Plasmodium falciparum* infection termed human cerebral malaria (CM) is estimated to account for three-quarters of the parasite's death toll (Brewster et al, 1990). Although not completely identical to the human disease, animal models have complemented clinical studies and *in vitro* experiments aimed at understanding the pathogenesis of CM. The most established of these is the infection of susceptible mice (*e.g*. C57BL and CBA backgrounds) with the ANKA strain of *Plasmodium berghei* (PbA). In this model of experimental cerebral malaria (ECM), at least 60% of susceptible mice develop neurological symptoms (ataxia, paralysis, head deviation, convulsions) culminating in coma and then death 6–12 days after inoculation with infected red blood cells (Engwerda et al, 2005). ECM is characterized by intravascular accumulation of infected red blood cells and leukocytes in the brain, petechial hemorrhages and breakdown of the blood–brain barrier (Thumwood et al, 1988).

Knockout mice have been instrumental in uncovering the cell types involved in ECM. Mice deficient in CD4^+^ T cells, CD8^+^ T cells, interferon-γ (IFN-γ) or its receptor are resistant to ECM, while B-cell-deficient mice remain susceptible (Amani et al, 2000; Yanez et al, 1996). The role of CD4^+^ T cells in C57BL/6 mice is restricted to the earlier induction phase of ECM, as antibody depletion of these cells prevented ECM if performed 4 days post-infection (p.i.) but not 6 days p.i.; in contrast, CD8^+^ T-cell depletion at the later time point, just 1 day before the onset of neurological symptoms, completely abrogated ECM death (Belnoue et al, 2002). It has recently been shown that IFN-γ production by CD4^+^ T cells recruits CD8^+^ T cells to the brain (Belnoue et al, 2008; Villegas-Mendez et al, 2012). Both perforin and Granzyme B (GrB) are essential for ECM, suggesting that damage to the blood–brain barrier may be a direct result of CD8^+^ T-cell cytolysis (Haque et al, 2011; Nitcheu et al, 2003).

Although considerable evidence implicates cytotoxic CD8^+^ T cells as the proximal cause of neuropathology in ECM, the specificities of these cells has remained a mystery. Studies with transgenic parasites bearing a model epitope from chicken ovalbumin confirmed that parasite-specific, brain-sequestered CD8^+^ T cells are indeed induced during infection (Lundie et al, 2008; Miyakoda et al, 2008). However, this immunodominant model epitope may not reflect immune responses against native malaria antigens. Further, such a transgenic system is not easily comparable to the human CM situation and hinders comparative studies between rodent malaria strains differing in their ability to induce ECM. Despite (or perhaps because of) the ∼5500 genes in *P. berghei*, not one CD8 epitope had yet been identified in C57BL/6 mice at the start of this work, prompting us to supply this deficiency.

**Figure 1:**
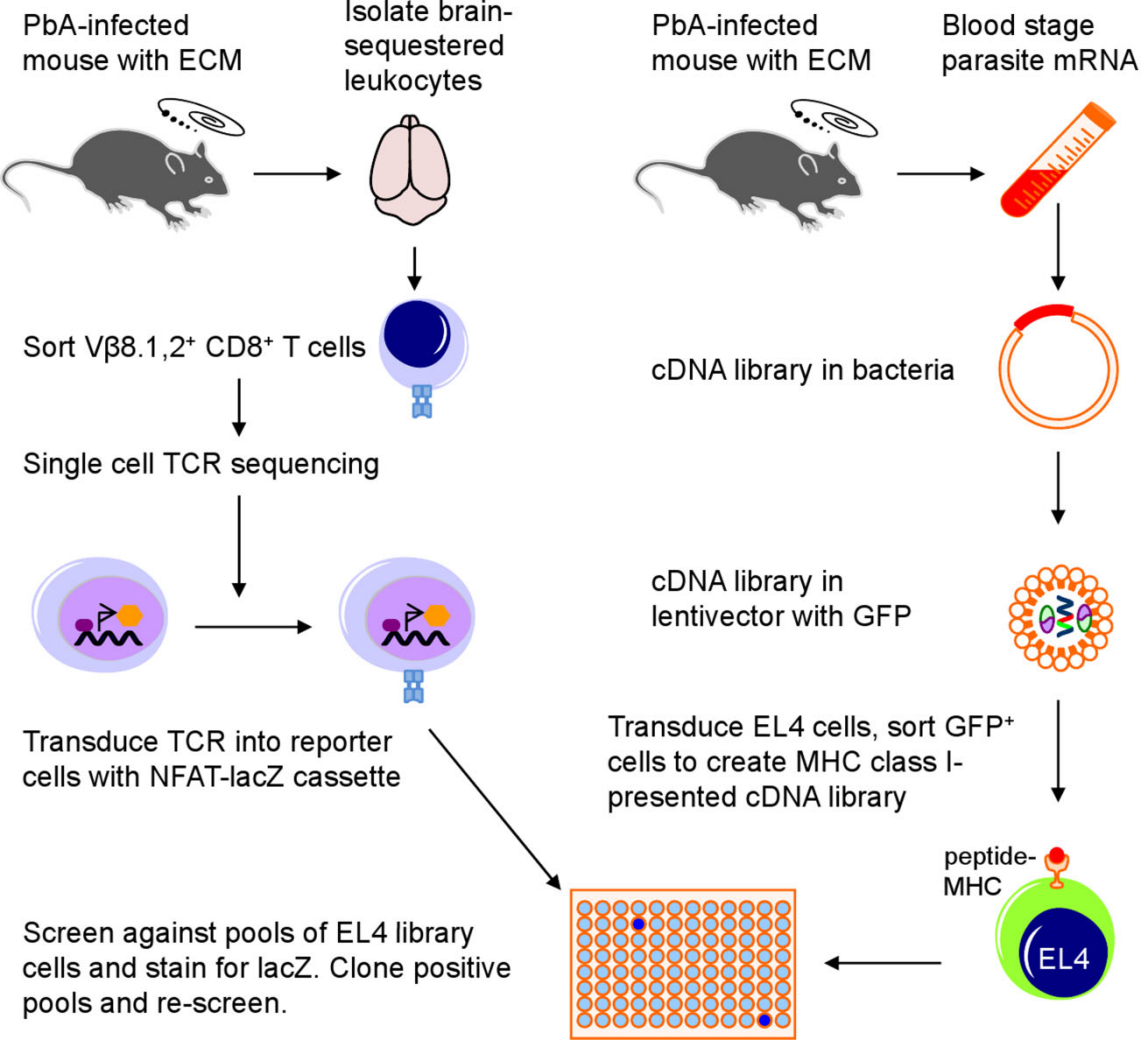
Schematic of antigen identification strategy Single cell TCR sequencing is performed on Vβ8.1,2^+^ CD8^+^ T cells sorted from the brains of PbA-infected C57BL/6 mice with ECM symptoms. The selected pair of TCR genes is transduced into a reporter cell bearing an NFAT-lacZ cassette. The reporter cells, LR-BSL8.4a, are used to screen a library of EL4 cells transduced to express fragments of PbA cDNA. Upon encountering the cognate peptide-MHC complex, the reporter cells express lacZ and are detected as blue spots following β-galactosidase staining. EL4 library cells from positive wells are cloned and re-screened to narrow down to single clones that are sequenced to identify the cognate antigen.

Our epitope identification strategy builds upon an established NFAT-*lacZ* reporter system for T-cell receptor (TCR) signalling (Sanderson & Shastri, 1994). Whereas the original approach fused T cells with partners bearing the NFAT-*lacZ* cassette, we sequenced TCR genes from individual T cells to select an over-represented pair to transduce into the reporter cells. By screening the TCR-transduced reporter cells against a library of antigen-presenting cells expressing PbA cDNA fragments, we sought to identify the cognate antigen in the library member/s able to induce *lacZ* expression (see schematic in Fig 1). To improve our chances of finding a highly immunogenic epitope, we focused our efforts on CD8^+^ T cells bearing the Vβ8 gene segment, which have been associated with ECM in susceptible mice (Belnoue et al, 2002; Boubou et al, 1999).

## RESULTS

### TCR sequencing of brain-sequestered CD8^+^ T cells reveals an over-represented motif

We sorted Vβ8.1,2^+^ CD8^+^ T cells from the brains of PbA-infected C57BL/6 mice exhibiting neurological signs and subjected these to single cell TCR sequencing. A clear motif emerged after a relatively small number of TCR genes were sequenced. Of 18 Vβ8.1 cells, 13 shared a “DWG” peptide sequence within the TCRβ junction (Table 1). These were paired with TCRα genes bearing a variety of Vα segments. Three cells from one mouse shared identical TCRα and β genes, indicating clonal expansion. We therefore selected this TCR pair to transduce into reporter cells bearing an NFAT-*lacZ* cassette, creating the LR-BSL8.4a cell line, so as to begin screening for the cognate antigen.

**Table 1:** Vβ8.1 TCR sequences derived from brain-sequestered CD8 T cells during ECM

Cell code	TRBV	TRBJ	TRBD	TCRβ junction	TRAV	TRAJ	TCRα junction
8.16	13-3	1-1	1	CASSRDRANTEVFF			
8.23	13-3	1-3	1	CASSDWGHSGNTLYF	17	57	CALEGRQGGSAKLIF
6.22	13-3	1-3	1	CASSEQGSGNTLYF	3-3	45	CAVSDAEGADRLTF
8.14	13-3	1-3	1	CASSERGSGNTLYF			
8.4	13-3	2-1	2	CASSDWGAGAEQFF	4D-4	52	CAAEANTGANTGKLTF
8.28	13-3	2-1	2	CASSDWGAGAEQFF	4D-4	52	CAAEANTGANTGKLTF
8.31	13-3	2-1	2	CASSDWGAGAEQFF	4D-4	52	CAAEANTGANTGKLTF
8.9	13-3	2-1	1	CASSLPGQGWAEQFF			
6.16	13-3	2-2	2	CASSDWGDTGQLYF			
8.6	13-3	2-4	2	CASSADWGGQNTLYF	4D-3	48	CAGGGNEKITF
6.23	13-3	2-4	2	CASSDWGSQNTLYF	8-2	42	CATVTGGSNAKLTF
6.29	13-3	2-5	2	CASSDWGGQDTQYF	8D-1	35	CATGG#TGFASALTF
6.2	13-3	2-5	2	CASSDWGNQDTQYF	16D	57	CAMSPQGGSAKLIF
8.15	13-3	2-5	2	CASSDWGNQDTQYF	14-2	39	GGAKLTF
6.30	13-3	2-5	2	CASSDWGQDTQYF			
6.1	13-3	2-5	2	CASSDWGTQDTQYF	12D-2	30	CALSDGTNAYKVIF
8.19	13-3	2-5	2	CASSDWGVQDTQYF	8D-1	56	CARPMATGGNNKLTF
6.17	13-3	2-7	1	CASSGTGTSSYEQYF			

Single-cell TCR sequencing was performed on Vβ8.1,2^+^ CD8^+^ T cells isolated from the brains of two mice 7 days after infection with PbA. Sequences were analyzed with IMGT/V-QUEST and only Vβ8.1 sequences (TRBV13-3 in IMGT notation) are reported here. Highlighted rows share a ‘DWG’ motif (underlined) in the TCRβ junction. TCRα sequences could not be obtained from some cells.

### Glideosome-associated protein 50 contains the cognate epitope

**Figure 2:**
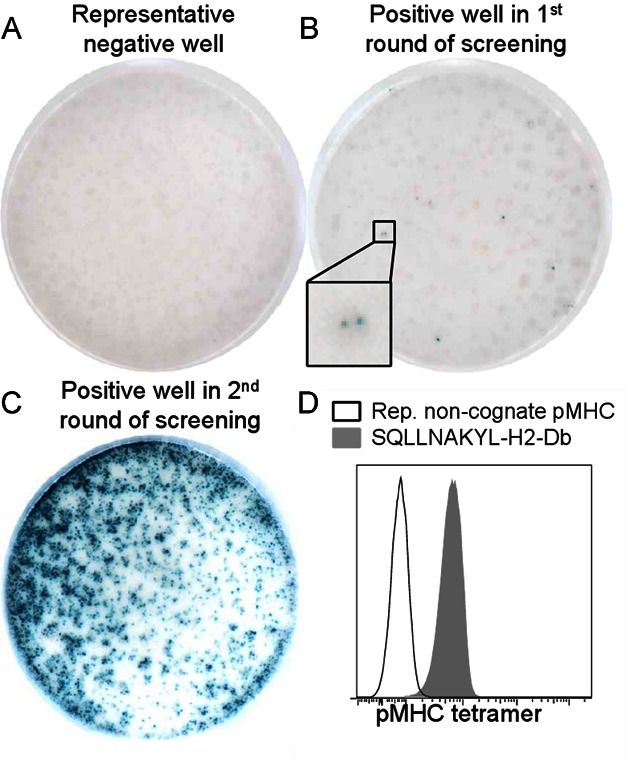
Identification of PbGAP50 as the antigen recognized by a Vβ8.1 TCR associated with ECM **A–C.** Well images of X-Gal-stained LR-BSL8.4a reporter cells incubated overnight with a library of EL4 cells transduced with *P. berghei* cDNA fragments. (A) A representative negative well. (B) The positive well in the first round of screening, containing about 250 library clones. An inset showing two blue cells at the original resolution is included. (C) A positive well in the second round of screening, containing a single library clone that was found to contain a fragment of PbGAP50.**D.** Predicted MHC epitopes in the fragment were used to generate peptide-MHC tetramers for staining LR-BSL8.4a cells. Open histogram: representative non-cognate peptide-MHC tetramer. Filled histogram: SQLLNAKYL-H-2D^b^ tetramer.

We created a library of EL4 cells (syngeneic for MHC genes with C57BL/6) expressing fragments of cDNA isolated from blood-stage PbA. Pools of the library cells were incubated with the LR-BSL8.4a TCR-transduced reporter cells in 96-well plates that were then stained for *lacZ* expression. One well was found to have 22 blue spots (*versus* a median of two in control wells, Fig 2). Individual clones from the positive library pool were screened again, resulting in three positive wells with thousands of blue cells (Fig 2). The positive EL4 clones were all sequenced and found to contain a fragment of *P. berghei* glideosome-associated protein 50 (PbGAP50, amino acids 40–119). Potential H-2D^b^ and K^b^ epitopes within this fragment were predicted with a computer algorithm and used to generate peptide-MHC tetramers by a rapid peptide-exchange strategy (Grotenbreg et al, 2008; Toebes et al, 2006). One tetramer was able to label LR-BSL8.4a cells, leading us to conclude that the TCR recognizes the H-2D^b^-restricted peptide SQLLNAKYL (Fig 2).

### SQLLNAKYL-specific brain-sequestering CD8^+^ T cells are induced during PbA infection

**Figure 3:**
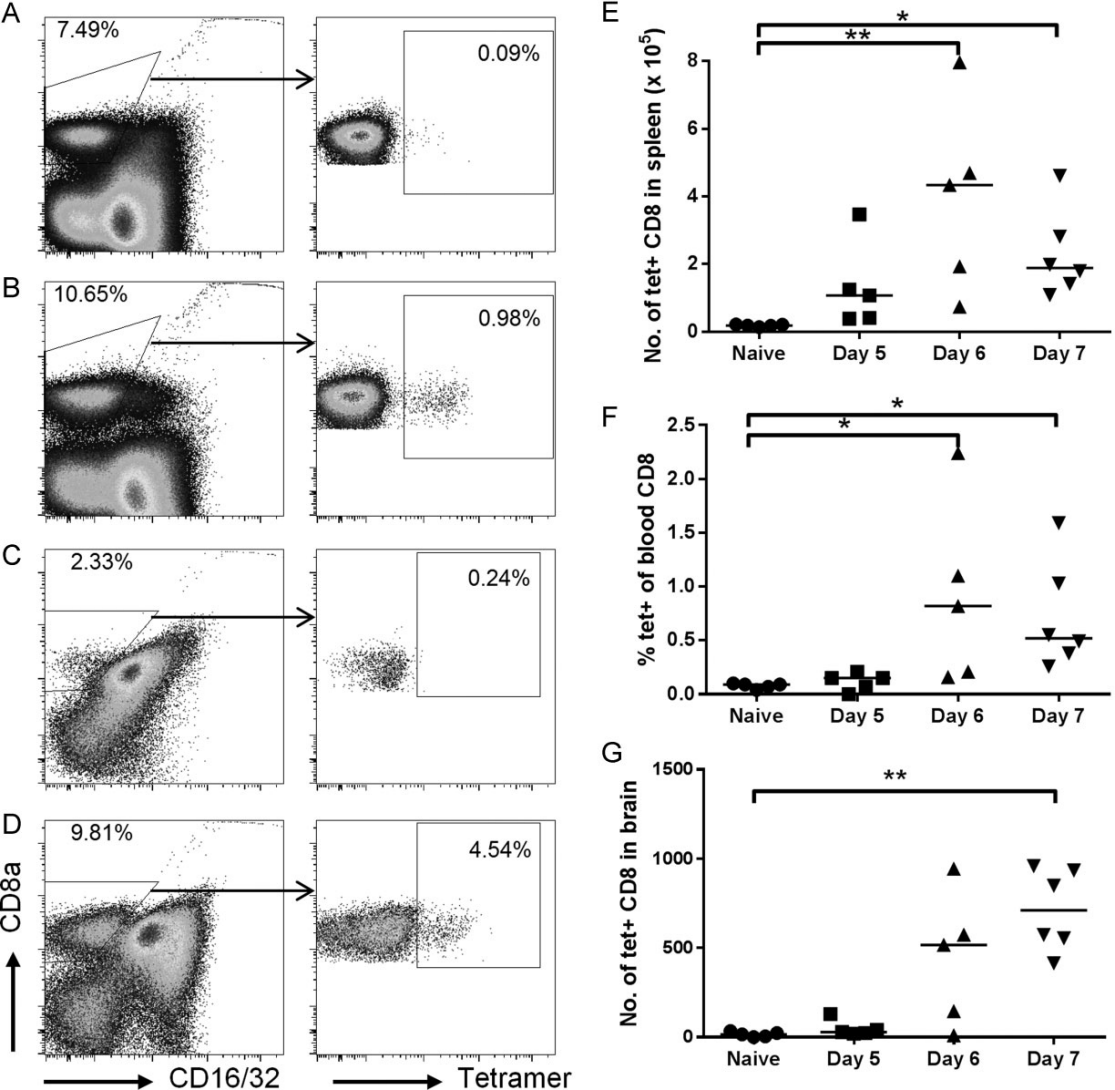
SQLLNAKYL-specific CD8^+^ T cells are induced during PbA infection and migrate to the brain just prior to the development of neurological signs SQLLNAKYL-H-2D^b^ tetramer staining was performed on splenocytes, blood and brain-sequestered leukocytes of naïve and PbA-infected mice 5, 6 and 7 days p.i. **A–D.** Representative dot plots of live cells, showing the gating of CD8^+^ T cells (CD8a^+^ CD16/32^−^) for tetramer analysis. (A) Naïve spleen. (B) Day 7 p.i. spleen. (C) Naïve brain. (D) Day 7 p.i. brain.**E–G.** Analysis of tetramer-labelled cells amongst CD8^+^ T cells in the spleen (E), blood (F) and brain (G). Bars represent medians. *p < 0.05, **p < 0.01, Kruskal–Wallis test with Dunn's post-test. Results are representative of three experiments.

Equipped with the SQLLNAKYL-H-2D^b^ tetramer, we set out to investigate the extent, kinetics and localization of the CD8^+^ T-cell response during PbA infection. Leukocytes from the spleens, blood and brains of naïve and infected C57BL/6 mice were subjected to tetramer staining, with CD8^+^ T cells identified by CD8 alpha staining and lack of CD16/32 staining (since CD8^+^ T ells lack the Fc receptors CD16 and CD32) (Fig 3). A distinct population of tetramer-positive cells (typically 0.5–2% of spleen CD8^+^ T cells and 2–9% of brain CD8^+^ T cells) was seen in infected but not naïve mice, validating the epitope specificity. Consistent with reports that the CD8 immune response in PbA is primed by splenic CD11c^hi^ Clec9A^+^ dendritic cells (deWalick et al, 2007; Piva et al, 2012), we saw that SQLLNAKYL-specific T cells appeared in some spleens as early as 5 days p.i. (Fig 3), whereas they expanded in the blood only from Day 6 onwards (Fig 3). These cells started migrating to the brain after 6 days but their numbers did not reach statistical significance until 7 days p.i. (Fig 3), a time when some mice started exhibiting neurological signs. On this day, there was no apparent correlation between the number of specific T cells in the brain and the clinical condition of the mice, suggesting that the localization of these cells in the brain precedes the development of ECM symptoms. Note that while SQLLNAKYL is predicted to bind to both H-2D^b^ and H-2K^b^ MHC molecules, the increase in tetramer staining of T cells during infection was seen only with the H-2D^b^ and not the H-2K^b^ tetramer.

### SQLLNAKYL-specific CD8^+^ T cells are functional and cytotoxic

**Figure 4:**
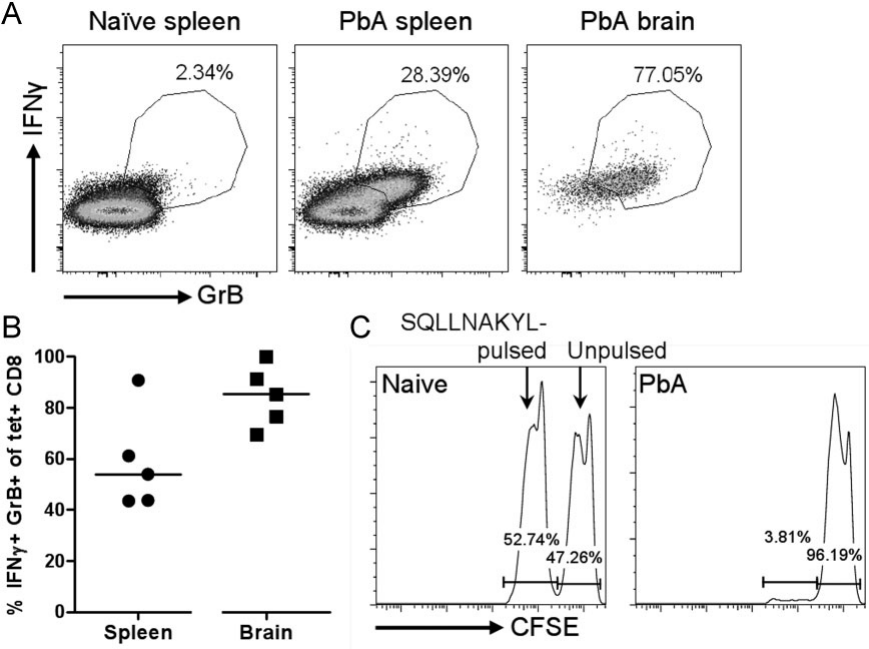
SQLLNAKYL-specific CD8^+^ T cells are functional and cytotoxic *in vivo* **A,B.** Combined tetramer and intracellular cytokine staining were performed on splenocytes and brain-sequestered leukocytes from PbA-infected mice 7 days p.i. Cells were incubated with Brefeldin A for 2 h without restimulation. (A) Representative IFN-γ and GrB profiles of live CD8^+^ T cells (CD8a^+^ CD16/32^−^). (B) The indicated IFN-γ^+^ GrB^+^ gate was used to analyze tetramer-labelled CD8^+^ T cells. Bars represent medians.**C.** Equal numbers of CFSE^hi^ unpulsed naïve splenocytes and CFSE^lo^ SQLLNAKYL-pulsed splenocytes were transferred into naïve or PbA-infected mice 6 days p.i. The mice were sacrificed 20 h later to analyze the CFSE-labelled cells in the spleens. The infected mouse is representative of *n* = 4, all with 96–97% specific lysis.

We further characterized the SQLLNAKYL-specific CD8^+^ T cells by combining MHC tetramer analysis with intracellular staining for IFN-γ and GrB. A population of IFN-γ^+^ GrB^+^ CD8^+^ T cells was present in the spleen 7 days p.i., and most of the brain-sequestered CD8^+^ T cells exhibited a similar cytokine production profile (Fig 4). A substantial proportion of the IFN-γ^+^ GrB^+^ CD8^+^ T cells were tetramer-positive: 3–9% in the spleen and 4–12% in the brain (Supporting Information Fig S1). No *ex vivo* restimulation had been performed, so these CD8^+^ T cells must have recently encountered their cognate peptide-MHC *in vivo*. At least 40% of the SQLLNAKYL-specific CD8^+^ T cells in the spleen and at least 70% in the brain were IFN-γ^+^ GrB^+^ (Fig 4), consistent with a role in ECM pathology. Since both perforin and GrB have been demonstrated to be necessary for ECM (Haque et al, 2011; Nitcheu et al, 2003), we examined whether SQLLNAKYL-specific CD8^+^ T cells had the ability to kill in an *in vivo* cytolysis assay. SQLLNAKYL-pulsed splenocytes transferred into PbA-infected mice were almost obliterated relative to unpulsed splenocytes (Fig 4). Taken together, these results point towards cytotoxic SQLLNAKYL-specific CD8^+^ T cells being in the right place at the right time to presumably damage the cells of the brain microvasculature.

### SQLLNAKYL-specific CD8^+^ T-cell response with non-ECM parasites

**Figure 5:**
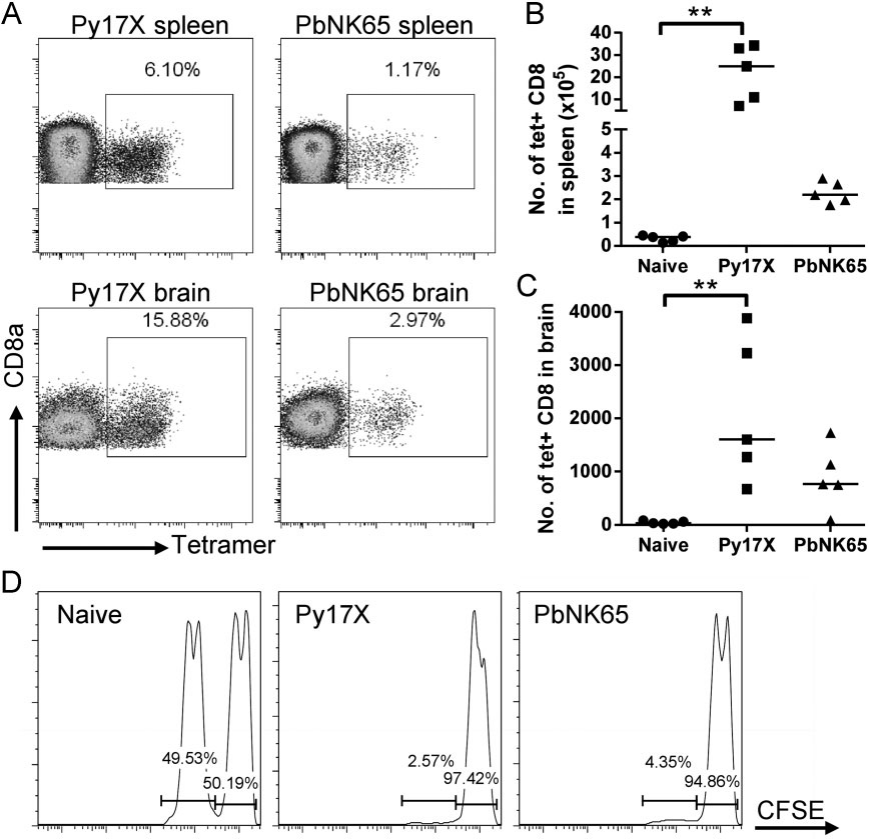
Cytotoxic SQLLNAKYL-specific CD8^+^ T cells are induced during infection with non-ECM-causing parasites **A,B.** SQLLNAKYL-H-2D^b^ tetramer staining was performed on splenocytes and brain-sequestered leukocytes of naïve, Py17X-infected and PbNK65-infected mice 7 days p.i.**A.** Representative dot plots of live CD8^+^ T cells (CD8a^+^ CD16/32^−^).**B,C.** Numbers of tetramer-labelled CD8^+^ T cells in the spleen (B) and brain (C). Bars represent medians. **p < 0.01, Kruskal–Wallis test with Dunn's post-test.**D.** Equal numbers of CFSE^hi^ unpulsed naïve splenocytes and CFSE^lo^ SQLLNAKYL-pulsed splenocytes were transferred into naïve, Py17X-infected or PbNK65-infected mice 6 days p.i. The mice were sacrificed 20 h later to analyze the CFSE-labelled cells in the spleens. Each infected mouse is representative of *n* = 4 (97–98% specific lysis for Py17X, 94–96% specific lysis for PbNK65).

While PbA induces ECM in C57BL/6 mice, there are other strains and species of rodent malaria that do not; determining why they differ is a key piece of the ECM etiology puzzle. The SQLLNAKYL epitope is conserved in the published genome sequence of *P. yoelii* 17X clone 1.1 non-lethal strain (Py17X; Carlton et al, 2002) and in the GAP50 protein of *P. yoelii* 17X clone YM lethal strain (PyYM) (PYYM_0822000). We sequenced the GAP50 gene in the NK65 strain of *P. berghei* (PbNK65) and found it to be identical to the PbA sequence. Py17X, PyYM and PbNK65 strains do not induce ECM, and we asked whether the CD8^+^ T-cell response against the SQLLNAKYL epitope could be missing or defective during infection with these strains, which could account for the lack of neuropathology. Surprisingly, MHC tetramer staining performed 7 days p.i. showed that not only were SQLLNAKYL-specific CD8^+^ T cells induced during infection with Py17X and PbNK65, they also accumulated in the brain (Fig 5). Indeed, the SQLLNAKYL epitope may be amongst the most immunodominant epitopes in Py17X, with the proportion of tetramer-labelled CD8^+^ T cells exceeding 15% in several brains (Fig 5). While the numbers of tetramer-labelled CD8^+^ T cells in the organs of PbNK65-infected mice did not reach significance in this non-parametric three-group analysis, they are comparable to those seen earlier with PbA (Fig 5). Furthermore, the specific CD8^+^ T cells were not cytolytically defective in mice infected with the non-ECM strains, as demonstrated in the *in vivo* cytotoxicity assay where transferred SQLLNAKYL-pulsed cells were killed (Fig 5).

### PbA but not non-ECM parasites induces brain vessel cross-presentation

**Figure 6:**
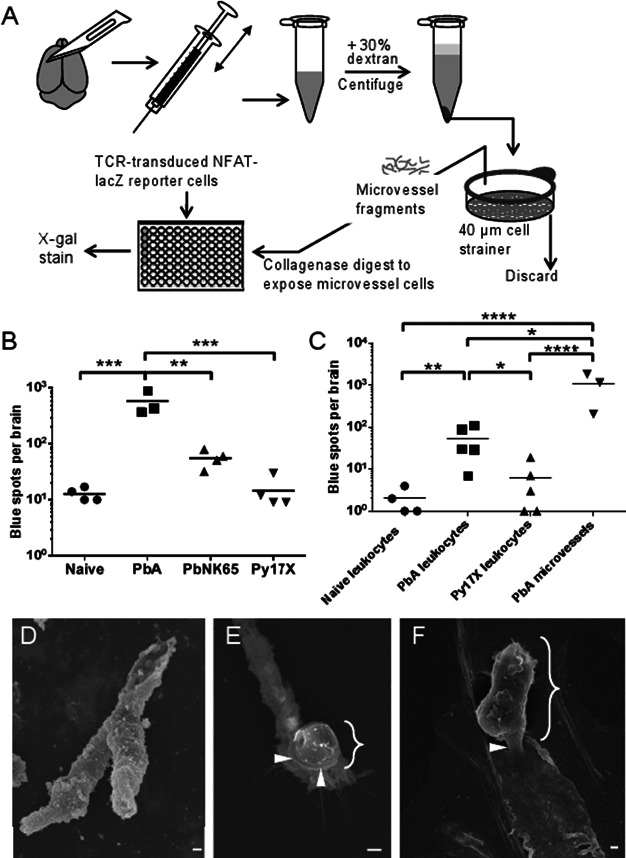
SQLLNAKYL is cross-presented by brain microvessels during PbA infection but not during infection by parasites that do not cause ECM **A.** Schematic of brain microvessel cross-presentation assay. Mouse brains were minced, homogenized through a needle and centrifuged with an equal volume of 30% dextran. The pellets were resuspended and passed over 40 μm cell strainers, and the retained microvessels were digested with collagenase and DNase I. After washing, the microvessel fraction from each brain was divided between five wells of a 96-well filter plate and co-incubated with LR-BSL8.4a cells overnight, prior to β-galactosidase staining.**B.** Brain microvessel cross-presentation results from naïve mice and mice 7 days post-infection with PbA, PbNK65 or Py17X. Bars represent means of 3–4 mice/group. **p < 0.01, ***p < 0.001, ANOVA with Bonferroni's post-test on log-transformed numbers. Results are representative of three experiments.**C.** Brain-sequestered leukocytes from naive mice and mice 7 days post-infection with PbA or Py17X were purified and co-incubated with LR-BSL8.4a cells overnight prior to β-galactosidase staining. The brain microvessel cross-presentation assay was performed on additional PbA-infected mice at the same time. Bars represent means of 3–5 mice/group. *p < 0.05, **p < 0.01, ****p < 0.0001, ANOVA with Bonferroni's post-test on log-transformed total spot counts arising from each brain.**D,E.** Scanning electron microscopy images of brain microvessel fragments from mice infected 7 days previously with PbA. Scale bars indicate 1 µm.**D.** Brain microvessel before collagenase digestion, not mixed with LR-BSL8.4a cells.**E.** Digested microvessel co-incubated with LR-BSL8.4a cells for 4 h.**F.** Digested microvessel co-incubated with LR-BSL8.4a cells for 24 h. Curly braces indicate LR-BSL8.4a cells and arrowheads indicate apparent cell–cell junctions LR-BSL8.4a cells have formed with microvessels.

**Figure 7:**
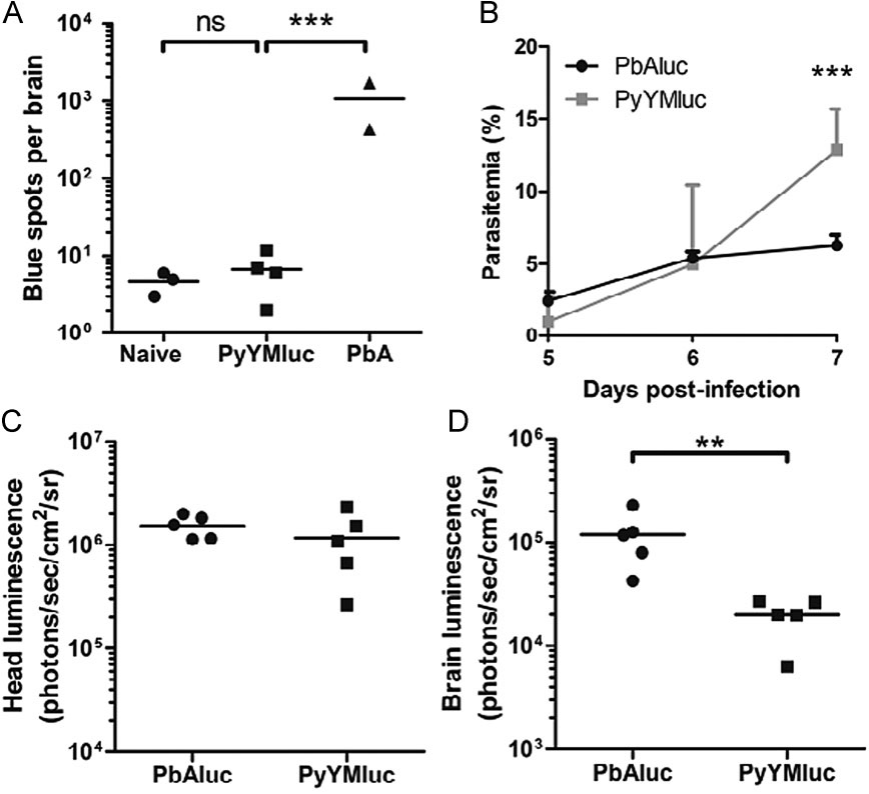
Cross-presentation of SQLLNAKYL is associated with parasite accumulation in the brain of infected mice **A.** Brain microvessel cross-presentation results from naïve mice and mice 7 days post-infection with PyYMluc or PbA. Bars represent means of 2–4 mice/group. ***p < 0.01, ANOVA with Bonferroni's post-test on log-transformed total spot counts arising from each brain.**B–D.** Parasitemia (B), head luminescence (C), and (D) luminescence of isolated and perfused brains from mice infected with PbAluc or PyYMluc. Parasitemia levels were determined at Days 5–7. Bars represent the mean ± SD. ***p < 0.01, Mann–Whitney test. Luminescence was measured at Day 7 post-infection. Bars represent the mean. **p < 0.01, *t*-test on values transformed with *x*′ = log(*x* + 1).

The observation that SQLLNAKYL-specific CD8^+^ T cells were not absent during infection with parasites that do not cause ECM prompted us to consider whether there was a lack of target cells for the cytolytic T cells to recognize. We and others have proposed that the brain endothelium becomes activated during infection, acquiring the ability to take up parasite material and cross-present parasite antigens, thus becoming targets for killing by specific CD8^+^ T cells (Belnoue et al, 2002; Nitcheu et al, 2003; Pino et al, 2005; Renia et al, 2006). We realized that the LR-BSL8.4a TCR-transduced reporter cells could potentially be used to detect such cross-presentation. Based on established protocols (Song & Pachter, 2003; Wu et al, 2003), we developed a technique to isolate microvessel fragments from homogenized mouse brains using dextran gradient centrifugation to remove the myelin-rich brain parenchyma tissue, followed by size fractionation to separate the vessel fragments from cells in suspension (Fig 6). Brain microvessels were isolated from naïve mice and mice infected 7 days earlier with PbA, PbNK65 or Py17X. After collagenase digestion of the vessel basal lamina, they were then incubated with LR-BSL8.4a cells overnight and stained for *lacZ* expression to determine if the reporter cells had encountered their cognate epitope. While the background staining from naïve mice amounted to only 10–20 blue spots, PbA-infected mice produced hundreds of blue spots (Fig 6), indicating the presence of SQLLNAKYL-presenting cells. In contrast, the numbers of blue spots resulting from PbNK65-, Py17X- (Fig 6) and PyYM-infected brains (Fig 7) were not significantly different from the naïve background and significantly lower than the PbA result.

Although these results suggest that the brain microvasculature cross-presents SQLLNAKYL only during PbA infection but not infection by non-ECM parasites, we needed to rule out the possibility that the presenting cells were actually contaminating myeloid cells that accumulate intravascularly and adhere in the lumen of the microvessels at the time of neurological signs. To do this, we isolated the brain leukocytes, including monocytes/macrophages and neutrophils (Belnoue et al, 2002) and tested them for the ability to trigger lacZ expression in LR-BSL8.4a cells. Although some cross-presentation was detected from the brain leukocytes of PbA-infected mice, the signal was less than one-tenth of the signal resulting from brain microvessels in the same experiment (Fig 6). Therefore, even if the microvessel preparations contain some myeloid cells, such cells cannot be the primary source of the detected cross-presentation. To investigate the role of myeloid cells in another way, we employed the MAFIA transgenic mouse model for macrophage/granulocyte depletion via a drug-inducible suicide gene (Burnett et al, 2004). MAFIA mice treated with the drug 5, 6 and 7 days p.i. were previously shown to have a ∼80% reduction of these myeloid cells in the blood and brain while remaining susceptible to ECM (Claser et al, 2011). We observed no reduction in brain microvessel cross-presentation in drug-treated MAFIA mice when compared to either untreated MAFIA mice or C57BL/6 mice (Supporting Information Fig S2A). Drug treatment even increased the number of blue spots, indicating that some myeloid cells may either compete for parasite antigen or regulate cross-presentation by the microvessels.

The role of collagenase digestion during the microvessel isolation protocol is to break down the basal lamina, allowing the exposed endothelial cells to protrude (Song & Pachter, 2003). When we omitted collagenase during the brain microvessel cross-presentation assay, the number of blue spots decreased by 80–90% (Supporting Information Fig S2B), suggesting that the cells responsible for cross-presentation may be those surrounded by the basal lamina, *i.e*. the endothelial cells or pericytes. We used scanning electron microscopy (SEM) to visualize interactions between the microvessels and LR-BSL8.4a cells. Note that both before (Fig 6) and after (Fig 6) collagenase digestion, the microvessels are in the form of multicellular tubes. After prolonged co-incubation (24 h in Fig 6), many of the vessels split open, presumably as endothelial cells migrate outwards. We have captured images of LR-BSL8.4a cells forming cell–cell junctions with both the outer surface of an intact vessel (Fig 6) as well as the lumenal surface of a split vessel (Fig 6). These cytoskeletal rearrangements may represent interactions akin to immunological synapses, with the cross-presenting cells being part of the microvessel walls. From these lines of evidence, we infer that cells constituting the blood–brain barrier, most probably endothelial cells, cross-present parasite antigens during PbA infection, thus becoming targets for CD8^+^ T-cell cytolysis. In PbNK65, Py17X and PyYM infection, on the other hand, the blood–brain barrier remains intact because there is little or no cross-presentation.

### Cross-presentation is associated with PbA parasite specific accumulation in the brain

Infected red blood cell accumulation in the brain capillaries have been recently strongly associated with the development of ECM (Amante et al, 2010; Baptista et al, 2010; Claser et al, 2011; McQuillan et al, 2011). We hypothesize that the difference in cross-presentation may reflect a difference in sequestration between ECM and non-ECM parasites. To test this, we used the ECM-inducing line PbAluc and the non-ECM-inducing line PyYMluc, which both possess the SQLLNAKYL epitope. Both lines are tagged with luciferase allowing us to assess parasite accumulation in deep tissues by measuring bioluminescence in the heads or brains isolated from infected mice after injection of the luciferase substrate (Supporting information Fig S3) (Claser et al, 2011). We first confirmed that microvessels from mice infected with the non-ECM line PyYMluc did not cross-present the SQLLNAKYL epitope in contrast to those from mice infected with PbA, the parental line of PbAluc (Fig 7). When parasite biological parameters were compared between PbAluc and PyYMluc, we observed that at the time of ECM signs for PbAluc (Day 7), PyYM parasitemia was higher than that of PbAluc and no difference in bioluminescence were detected when the heads of the infected animals were imaged (Fig 7). However, bioluminescence in the perfused brains of infected mice was one log lower with PyYMluc compared to PbAluc (Fig 7). This suggests that local accumulation of PbA-infected red blood cells may lead to increased contact to and/or cytoadherence to brain microvessels, facilitating antigen uptake required for cross-presentation.

### SQLLNAKYL-specific CD8^+^ T cells damage the blood–brain barrier

**Figure 8:**
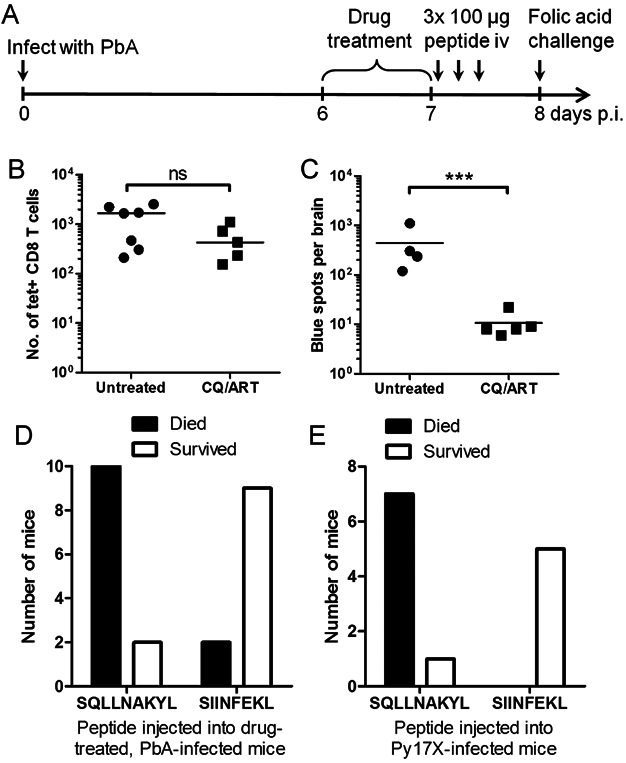
SQLLNAKYL-specific CD8^+^ T cells damage the blood–brain barrier **A.** Experiment schedule for (D). Mice infected with PbA were treated with chloroquine and artesunate (CQ/ART) between 6 and 7 days p.i., then injected three times i.v., 4 h apart with 100 µg of either SQLLNAKYL peptide or control SIINFEKL peptide. They were injected with folic acid 8 days p.i. to test the integrity of the blood–brain barrier.**B,C.** Mice infected with PbA were untreated or treated with CQ/ART between 6 and 7 days p.i., then sacrificed soon after.**B.** Tetramer staining was performed on the brain-sequestered leukocytes. Bars represent medians. ns, not significant by Mann–Whitney test.**C.** Microvessels were isolated from each brain and co-incubated with LR-BSL8.4a cells overnight. The total number of blue spots after β-galactosidase staining is reported. Bars represent means. ***p < 0.001, Student *t*-test on log-transformed data.**D.** Mice subjected to the protocol in (A) either convulsed and died within 90 min or survived until the next day. *p* = 0.0033, Fisher's exact test.**E.** Mice infected with Py17X were injected i.v. with 100 µg of either SQLLNAKYL or SIINFEKL peptide 7 days p.i., then challenged with folic acid 6 h later. *p* = 0.0047, Fisher's exact test.

Are the SQLLNAKYL-specific CD8^+^ T cells able to compromise the blood–brain barrier by killing endothelial cells presenting the epitope? To support this model, we devised an experiment to demonstrate that SQLLNAKYL-specific CD8^+^ T cells contribute to the neuropathology seen during ECM. Two groups reported that treatment with anti-malarial drugs 1 day before ECM is expected prevents the development of neurological signs even though CD8^+^ T cells still accumulate in the brain (Baptista et al, 2010; Haque et al, 2011). We postulated that anti-malarial drug treatment would severely reduce the amount of parasite antigen available in the brain to be cross-presented by the microvasculature, thus preventing ECM. However, if the microvasculature was then exposed to soluble SQLLNAKYL peptide via repeated intravenous (i.v.) injection (see schedule in Fig 8), then the specific CD8^+^ T cells present in the brain vasculature would presumably be able to recognize the peptide loaded onto class I MHC molecules.

First, we investigated the effects of combined chloroquine and artesunate drug treatment for a 24-h period starting 6 days p.i. with PbA. Peripheral parasitemia levels dropped by an order of magnitude after treatment, and as expected, the parasite biomass in the brain (as measured by *in vivo* imaging of PbAluc in a separate experiment) was likewise reduced (Supporting Information Fig S4). This treatment also protected all mice from developing ECM. As expected, immediately following the treatment, there was no significant change in the number of SQLLNAKYL-specific CD8^+^ T cells in the brain compared to untreated mice (Fig 8). Conversely, there was a drastic reduction in brain microvessel cross-presentation of the SQLLNAKYL epitope, down to essentially background levels (Fig 8). In an additional control experiment, we verified that reduced endogenous presentation of the SQLLNAKYL epitope was not due to an inhibition of MHC class I expression by the drugs used to clear the parasites. Indeed, chloroquine has been known to affect MHC molecules (Belizaire & Unanue, 2009; Kurotaki et al, 2007). Thus, we devised a modified presentation assay where the peptide SIINFEKL corresponding to the major CD8 epitope of chicken ovalbumin was used to pulse brain microvessels before mixing together with a reporter cell line recognizing the OVA peptide in the context of MHC class I molecule. We chose this peptide over the SQLLNAKYL peptide to eliminate any cofounding factor due to endogenous presentation of the SQLLNAKYL peptide. We observed that exogenous addition of the OVA peptide to the microvessels from untreated PbA-infected mice induces similar numbers of blue spots compared to those from chloroquine/artesunate-treated infected mice, with both groups giving a much higher response than naive mice (Supporting Information Fig S5). This means that more MHC class I molecules are expressed by brain microvessels after PbA infection and drug treatment did not affect MHC class I expression. All together, this indicates that *in situ* parasite load controls parasite antigen presentation and is a key factor for ECM to develop.

To further investigate whether CD8^+^ T-cell recognition of SQLLNAKYL epitope presented in the brain *in vivo* contributes to ECM pathology, SQLLNAKYL peptide or the irrelevant SIINFEKL peptide (the immunodominant epitope from ovalbumin in C57BL/6 mice) was injected i.v. after drug treatment. We observed that SQLLNAKYL-treated mice (eight out of nine) displayed early signs of ECM (ruffled fur, hunching and lethargy) but did not progress to paralysis or coma whether or not additional peptide injections were administered. None of the eight mice treated with the control peptide exhibited these signs. On hindsight, the mild pathology was not surprising because SQLLNAKYL-specific CD8^+^ T cells accounted for only 4–12% of the IFN-γ^+^GrB^+^ CD8^+^ T cells in the brain (Supporting Information Fig S1). Nevertheless, we sought a more sensitive and objective method of detecting damage to the blood–brain barrier and adopted the approach of Hermsen et al, who intravenously injected folic acid into mice infected with *P. berghei* K173. In C57BL/6 mice infected with this parasite, the neurotoxin induced convulsions and death within 90 min of injection, whereas an intact blood–brain barrier protected uninfected mice (Hermsen et al, 1998). We performed the folic acid challenge 8 days p.i. on PbA-infected mice that had undergone drug treatment between 6 and 7 days p.i., followed by three injections of SQLLNAKYL or control peptide (Fig 8). Ten out of 12 SQLLNAKYL-treated mice convulsed and died, in contrast to only 2 out of 11 mice given the control peptide (Fig 8), a significant difference (*p* = 0.0033 by Fisher's exact test) indicating a relative risk of 4.58. We therefore conclude that CD8^+^ T cells recognizing the SQLLNAKYL epitope are able to damage the blood–brain barrier *in vivo* and thus are likely to contribute to ECM pathology.

Our earlier results indicated that Py17X infection induces a strong, cytotoxic CD8 response against SQLLNAKYL (Fig 5), but the lack of target cells presenting this epitope in the brain microvasculature could explain the absence of ECM. We asked if we could induce ECM-like pathology in Py17X-infected mice by pulsing the brain microvasculature with SQLLNAKYL peptide. Six hours following a single SQLLNAKYL i.v. injection 7 days p.i., the mice displayed early signs of ECM and seven out of eight died of convulsions after folic acid challenge (Fig 8). In contrast, all the SIINFEKL-injected control mice (*n* = 5) had no such symptoms and survived the folic acid injection (*p* = 0.0047, Fisher's exact test). These results reinforce our proposition that brain microvessel cross-presentation of parasite antigens is a key step in ECM pathogenesis and distinguishes ECM-inducing parasites from those that do not.

## DISCUSSION

Investigations of ECM etiology have been hampered by the lack of known CD8^+^ T-cell epitopes, motivating us to tackle the task of epitope identification. To do so, we developed two essential complimentary tools, a *P. berghei* antigen library in EL4 cells suited for class I MHC haplotype H-2^b^ presentation and a reporter cell line, LR-BSL8.4a, that expresses α and β chains of the TCR derived from FACS-sorted, clonally expanded brain-sequestered T cells. Screening of the library pinpointed a fragment of GAP50 as being the cognate antigen of this TCR, in turn allowing us to identify the SQLLNAKYL epitope. Shortly after we identified this CD8^+^ T epitope, Lau and colleagues reported five other peptides that induced IFN-γ secretion in up to 1% of splenic CD8^+^ T cells from PbA-infected mice (Lau et al, 2011). Unfortunately, they were unable to generate MHC tetramers at that time, limiting further characterization to just *in vivo* cytotoxicity. Moreover, they did not demonstrate whether CD8^+^ T cells recognizing these epitopes are able to damage the blood–brain barrier and mediate ECM. In this work, we have developed a powerful toolkit for probing ECM pathogenesis, consisting of the SQLLNAKYL peptide, the corresponding peptide-MHC tetramer and the LR-BSL8.4a reporter cell line, allowing us to study not just the specific CD8^+^ T cells but also cross-presentation in the brain. SQLLNAKYL-specific T cells accounted for 4–12% of the activated CD8^+^ T cells in the brain. The true extent of the CD8^+^ immune response against this epitope may be even higher than estimated by MHC tetramer staining since low affinity TCRs may not be labelled. For instance, reporter cells transduced with another motif-containing Vβ8.1 TCR (cell 6.2 in Table 1) expressed *lacZ* when incubated with SQLLNAKYL-pulsed cells but were MHC tetramer-negative.

Through tetramer labelling, intracellular cytokine staining and *in vivo* cytotoxicity assays, an incriminating picture of SQLLNAKYL-specific CD8^+^ T cells emerged. They had the means (GrB^+^ and cytotoxic) and opportunity (brain sequestration 7 days p.i.) to be responsible for the neuropathology seen during PbA infection. We performed several experiments to adoptively transfer SQLLNAKYL-specific CD8^+^ T cells (either sorted from infected mice or from a generated CD8^+^ T-cell line) into CD8-deficient mice but they were invariably deleted following PbA infection. We thus provided evidence for the ability of SQLLNAKYL-specific CD8^+^ T cells to damage the blood–brain barrier by rapidly clearing PbA antigens with anti-malarial drugs, then introducing soluble SQLLNAKYL peptide into the circulation that can be loaded onto MHC molecules presented by brain microvessel cells *in vivo*. One limitation of our approach is that blood–brain barrier integrity was assessed by a very sensitive assay, by injecting folic acid intravenously. Folate has a direct epileptogenic effect on neurons, causing convulsions and death if it can access the central nervous system via a breach in the blood–brain barrier (Hommes & Obbens, 1972; Obbens & Hommes, 1973). Without performing the folic acid assay, the damage caused by CD8^+^ T cells of just this one specificity was insufficient to recapitulate the full extent and range of pathologies of ECM.

The presence of cytotoxic SQLLNAKYL-specific CD8^+^ T cells alone does not ensure neurological damage, since such cells were also found in mice infected with PbNK65 and Py17X, which do not cause ECM. To take the criminal analogy further, we then turned to the question of the “motive” for killing, which in CD8^+^ T cells generally means interaction with cells expressing the cognate peptide-MHC complex. Could a lack of cross-presenting “victim” cells in the brain microvasculature during infection with PbNK65, Py17X and PyYM account for the failure to induce ECM? We devised a novel brain microvessel cross-presentation assay and saw that the SQLLNAKYL epitope is presented by the cerebral microvasculature during PbA but not PbNK65 and Py17X infection. It remains to be seen whether the absence of cross-presentation can be generalized to all parasite antigens, and if so, which aspects of parasite biology or host response account for this difference. Parasite load in the brain was markedly reduced in PbNK65 compared to PbA infection (Baptista et al, 2010), so a simple deficiency of parasite material available for processing locally in the brain during the first week of infection is part of the answer. This was confirmed by using luciferase-tagged PbA and PyYM. PyYMluc had more circulating parasites than PbAluc, but much fewer PyYM-infected red blood cells accumulated in the brain following perfusion than those of PbA. This lower accumulation in the brain was associated with an absence of cross-presentation of the SQLLNAKYL epitope by microvessels of PyYMluc-infected mice. Our data also suggest that parasites that are merely circulating in blood vessels in the brain may not be phagocytosed by brain microvessel cells. Nevertheless, we have demonstrated that treatment with chloroquine and artesunate massively reduces the parasite load in the periphery and more importantly in the brain, preventing antigen presentation and ensuing ECM death.

We have provided the first evidence of malaria parasite cross-presentation by brain microvessel cells. PbA parasites do not infect the brain parenchyma. However, a fraction of PbA-infected red blood cells accumulate intravascularly (Amante et al, 2010; Claser et al, 2011) and cytoadhere to endothelial cells (unpublished results). It is thus likely that endothelial cells are acting as antigen-presenting cells (Razakandrainibe et al, 2012; Renia et al, 2006). As shown previously, endothelial cells in retinal wholemounts undergo apoptosis via a perforin-dependent pathway during ECM, suggesting that brain CD8^+^ T cells specifically recognize parasite-derived peptide-MHC complexes on endothelial cells (Potter et al, 2006). Endothelial cells from a number of organs have been shown to be capable of cross-presentation, including the liver, pancreas, aorta and lymph node (Bagai et al, 2005; Limmer et al, 2000; Lund et al, 2012; Savinov et al, 2003). Recently, a non-canonical, TNF-mediated mechanism by which CD8^+^ T cells can kill cross-presenting endothelial cells was discovered (Wohlleber et al, 2012). This mechanism is unlikely to play a major role in ECM since TNF-α-deficient mice remain susceptible to ECM (Engwerda et al, 2002), whereas IFN-γ (Amani et al, 2000), perforin (Nitcheu et al, 2003) and Granzyme B (Haque et al, 2011) are all required for ECM.

In our protocol, the brain microvessel fragments were isolated without relying on any molecular markers, and even after collagenase digestion, the multicellular tubes preclude analysis or sorting by flow cytometry. More extensive digestion to yield a single cell suspension greatly decreases viability (Song & Pachter, 2003). Therefore, while endothelial cells are the major cell type present in the microvessel preparation, we cannot yet conclude definitively that these cells are responsible for the detected cross-presentation. Nevertheless, we have ruled out contaminating myeloid leukocytes as being the major cross-presenting population, since when brain leukocytes are intentionally purified, they stimulate the reporter cells an order of magnitude less than brain microvessels. Furthermore, we observed no decrease in brain microvessel cross-presentation from MAFIA mice when they were treated to deplete most of the macrophages and granulocytes. In addition to endothelial cells and pericytes that are surrounded by basal lamina, blood vessels in the brain are associated with astrocyte foot processes and microglia. The observation that omitting collagenase digestion of the basal lamina greatly decreases the detected microvessel cross-presentation argues against astrocytes and microglia being responsible, as do SEM images of reporter cells interacting with both the ablumenal and lumenal surfaces of vessel walls. Further studies using the TCR-transduced reporter cells to decipher whether and how endothelial cells cross-present are underway. This is of importance since elegant studies show that endothelial cells from other organs, such as the liver, cross-present antigens using different mechanisms, kinetics and dynamics of antigen uptake compared to dendritic cells (Kurts et al, 2010; Schurich et al, 2009).

The relevance of the murine ECM model to human CM has been disputed, but we and others contend that understanding the mechanism of ECM pathogenesis suggests lines of inquiry for investigating human disease (Craig et al, 2012; Renia et al, 2010). Naturally acquired CD8^+^ immune responses against several *P. falciparum* proteins have been detected (Chelimo et al, 2011; Dodoo et al, 2011; Woodberry et al, 2009), and it would be informative to find out if GAP50 is also immunogenic in humans. While the pathogenic role of CD8^+^ T cells has been clearly demonstrated in ECM, evidence of their involvement in human CM is lacking, with the main criticism being their absence or rarity in post-mortem histology samples (Renia et al, 2012). However, even in an ECM-afflicted mouse brain with a volume of about 400 mm^3^, there may only be a total of 50,000 CD8^+^ T cells following perfusion or exsanguination, implying that on average, six 500 µm square, 5 µm thick sections would have to be examined to find a single CD8^+^ T cell. Techniques more sensitive than histology are definitively required to detect malaria-specific CD8^+^ T-cell accumulation in human brains. On the other hand, our work suggests an alternative approach for investigating whether CD8^+^ T-cell cytolysis plays a role in human CM, which is to search for evidence of brain microvessel cross-presentation of parasite antigens. Human brain endothelial cells cultured *in vitro* with *P. falciparum* parasites upregulate many genes associated with inflammation and the immune response, including Antigen Peptide Transporter 1 (TAP1) and class I HLA molecules (Tripathi et al, 2009), suggesting increased cross-presentation capability. Parasite engulfment and transfer of parasite antigens to endothelial cell endosomes have also been observed in co-culture experiments (Jambou et al, 2010). It remains to be established if parasite-derived epitopes in the context of class I MHC molecules can be detected on endothelial cells *in vitro* or even in post-mortem brain tissue, perhaps by peptide elution (Fissolo et al, 2009) or using a reporter cell strategy such as we used here. Since human CM is characterized by adhesion and sequestration of infected red blood cells in brain capillaries, the local concentration of parasite material available for cross-presentation could well be higher than in the murine model. Based on our data, we propose that fast-acting drugs able to decrease parasite load *in vivo* should be used to prevent the antigen presentation and ensuing lethal cascade.

## MATERIALS AND METHODS

### Mice

C57BL/6J female mice (5–8 weeks old) were used unless otherwise stated. Macrophage Fas-induced Apoptosis (MAFIA) mice, which bear an inducible suicide gene under the *Csf1r* promoter (Burnett et al, 2004), were treated with the dimerizer drug AP20187 as previously described (Claser et al, 2011). The mice were bred under specific pathogen-free conditions in the Biomedical Resource Centre, Singapore. All animal experiments and procedures were approved by the Institutional Animal Care and Use Committee (IACUC) and complied with the guidelines of the Agri-Food and Veterinary Authority (AVA) and the National Advisory Committee for Laboratory Animal Research (NACLAR).

### Parasites and infection

Five *Plasmodium* lines were used: *P. berghei* ANKA clone 15Cy1 (PbA), a GFP-luciferase-transgenic derivative of PbA clone 15Cy1 (Franke-Fayard et al, 2005) (PbAluc), *P. berghei* NK65 (PbNK65) uncloned line (Yoeli & Most, 1965), *P. yoelii yoelii* 17XNL clone 1.1 (Py17X; Weiss et al, 1989), and a GFP-luciferase-transgenic derivative of *P. yoelii yoelii* clone YM (PyYMluc; Mwakingwe et al, 2009). Mice were infected by intraperitoneal injection of 10^6^ infected red blood cells, from stabilates prepared by passage in C57BL/6J mice and stored in liquid nitrogen in Alseveer's solution. In some experiments, parasitemia was determined by flow cytometry (Malleret et al, 2011).

### Creation of cDNA library

*P. berghei* blood stage cDNA fragments (average ∼400 bp) were produced by random priming with phosphorothioate-modified primers as we previously described (Howland et al, 2011). A total of 1.2 × 10^5^ clones enriched for in-frame inserts were produced by in-fusion cloning into a specially constructed plasmid. The inserts were then transferred into a lentiviral transfer plasmid based on pWPXL (kindly provided by Dr. Didier Trono, Ecole Polytechnique Fédérale de Lausanne, Switzerland), downstream of a GFP gene and a 2A self-cleaving peptide. Details of the plasmids and lentivector production are included in the Supporting Information. Lentiviral particles were produced and used to transduce EL4 cells (ATCC). GFP-expressing EL4 cells were sorted, expanded and cryopreserved to constitute the cDNA library.

### Leukocyte isolation

Mice were terminally exsanguinated retro-orbitally under ketamine/xylazine anesthesia before the brains and spleens were removed. We have not observed a significant difference in CD8^+^ T-cell numbers in the brain of infected mice between exsanguinated and perfused mice. Heparinized blood was treated twice with ACK lysis buffer to remove red blood cells. Spleens were mashed, passed through a 40 µm cell strainer and subjected to ACK lysis. Each brain was mashed, digested for 30 min at room temperature with 0.5 mg/ml collagenase type 4 (Worthington) and 10 µg/ml DNase I (Roche) in 10 ml PBS, and then passed through a 40 µm cell strainer. After a brief centrifugation to remove large debris, the cells were centrifuged at 1900*g* for 10 min over a 30% Percoll gradient. The cells in the pellet were treated with ACK lysis buffer and washed.

### Single cell TCR sequencing

Brain-sequestered leukocytes from PbA-infected mice displaying neurological signs were labelled with αCD8α-APC, αVβ8.1,2-FITC (BD Biosciences) and DAPI. Live double-positive cells were sorted singly into PCR tubes containing the reaction buffer. We adapted a published protocol (Ozawa et al, 2008) of single cell human TCR sequencing for mouse cells; further details are included in the Supporting Information. The sequences were analyzed using IMGT/V-QUEST (Brochet et al, 2008).

### Generation of TCR-transduced reporter cells

Variable regions from the brain-sequestered leukocyte cell 8.4 were assembled by PCR with the constant regions into a single open reading frame, with the two chains separated by a 2A self-cleaving peptide. LR-BSL8.4a cells were cloned after lentivector transduction of these TCR genes into LR-Ø cells bearing an NFAT-*lacZ* cassette (see Supporting Information).

### Library screening

EL4 cells transduced with the PbA cDNA library were seeded at 250 cells/well in 96-well plates and allowed to grow up. About 3 × 10^4^ library cells from each well were transferred to 96-well filter plates (Pall 8029) and co-incubated with 3 × 10^4^ LR-BSL8.4a cells overnight. The plates were then stained for 6 h with X-gal as published (Sanderson & Shastri, 1994), with solution changes accomplished by centrifuging the plates briefly to drain them. Blue spots were imaged and counted on a CTL ImmunoSpot Analyzer. Library cells giving rise to the single positive well (out of 11 plates) were cloned by sorting; the clones were then screened in the same manner.

**The paper explained**

PROBLEM:

CM, a severe neurological complication of infection by *P. falciparum*, is the major cause of malaria mortality but remains poorly understood. A murine model of CM using *P. berghei* ANKA (PbA) infection represents a valuable tool for deciphering the mechanisms of neurological damage, which involve both host and parasite contributions. CD8^+^ T cells have been demonstrated to play an effector role in ECM. However, the lack of known CD8^+^ T-cell epitopes has impeded further understanding of ECM pathogenesis. One unanswered question is what differentiates PbA from other rodent *Plasmodium* parasites that do not cause ECM.

RESULTS:

To discover the cognate antigen of a population of CD8^+^ T cells sequestered in the mouse brain during ECM, we created and screened a PbA cDNA library expressed in antigen-presenting cells using a TCR-transduced reporter cell line. We thus identified a class I MHC epitope in glideosome-associated protein 50 that elicits a strong CD8^+^ T-cell response during PbA infection. Unexpectedly, we found that cytotoxic, brain-migrating CD8^+^ T cells recognizing this conserved epitope are also induced during infection with *Plasmodium* parasites that do not cause ECM. However, using the TCR-transduced reporter cell line, we determined that this peptide-MHC complex was presented on brain microvessels isolated from PbA-infected mice, but not from mice infected with the non-ECM-causing parasites. These results support a model of ECM pathogenesis where brain endothelial cells cross-presenting PbA-derived epitopes become targets of CD8^+^ T-cell-mediated cytolysis, leading to disruption of the blood–brain barrier. We further showed that fast-acting drugs that reduce the parasite load *in vivo* reduce presentation and prevent ECM death.

IMPACT:

The epitope, peptide-MHC tetramer and TCR-transduced reporter cell line developed in this work constitute a powerful tool kit for further mechanistic studies of ECM pathogenesis. The discovery that brain microvessel cross-presentation differentiates PbA from non-ECM-causing parasites has important implications for human CM, which develops in only a small fraction of infected children. Interventions targeting parasite accumulation in the brain and/or brain endothelial cell cross-presentation pathways have potential therapeutic value.

### Epitope identification

Potential H-2K^b^ and H-2D^b^ epitopes were predicted and used to produce PE-labelled tetramers by peptide exchange as previously described (Grotenbreg et al, 2008). Malaria peptides were obtained from Genscript while the SIINFEKL peptide from ovalbumin was obtained from Mimotopes. LR-BSL8.4a cells were labelled with each tetramer and analyzed by flow cytometry.

### Tetramer staining

Spleen, blood or brain leukocytes were first stained with LIVE/DEAD Violet (Life Technologies). Next, they were incubated with PE-labelled SQLLNAKYL-H-2D^b^ tetramer for 15 min on ice before αCD8a-APC (BD) and αCD16/32-APC-Cy7 (Biolegend) were added. After 30 min incubation on ice, the cells were washed and fixed in 1% formaldehyde. For brains, the entire sample was acquired on a MACSQuant Analyzer and the number of live CD8^+^ CD16/32^−^ tetramer^+^ cells is reported directly. For spleens, the number of cells in this sub-population was calculated from the total splenocyte count.

### Intracellular cytokine staining

Spleen and brain leukocytes were cultured in medium containing 10 µg/ml Brefeldin A for 2 h before tetramer staining was performed, substituting LIVE/DEAD Aqua and αCD8a-PerCP-Cy5.5 (Biolegend). After overnight fixation in 2% formaldehyde at 4°C, the cells were permeabilized using 0.5% saponin and stained with αIFN-γ-FITC (BD) and αGranzymeB-PE-Cy7 (eBiosciences) for 20 min at room temperature.

### *In vivo* cytolysis assay

Naïve splenocytes were divided into two portions. One portion was incubated with 10 µg/ml SQLLNAKYL peptide for 1 h at 37°C, then washed and labelled with 0.5 µM CFSE for 10 min at 37°C; the other was not pulsed with peptide and labelled with 5 µM CFSE. Equal numbers of peptide-pulsed and unpulsed splenocytes (10^7^ cells each) were injected i.v. into naïve mice or mice infected 6 days previously. The mice were sacrificed 20 h later to analyze the CFSE-labelled cells in the spleen.

### Brain microvessel cross-presentation assay

The technique for isolating brain microvessels was adapted from published protocols (Song & Pachter, 2003; Wu et al, 2003). Each anesthetized mouse was terminally bled before the brain (without the meninges and brain stem) was finely minced with 1 ml of medium and homogenized by passing five times through a 23-gauge needle. The homogenate was mixed with an equal volume of 30% dextran (MW ∼70,000, Sigma–Aldrich) in PBS and centrifuged at 10,000*g* for 15 min at 4°C. The pellet was resuspended in PBS and passed through a 40 µm cell strainer that retains the microvessels. After washing, the cell strainer was back-flushed with 2 ml PBS over a 6-well plate to collect the microvessels, which were rocked at room temperature with 2% foetal bovine serum, 1 mg/ml collagenase 4 and 10 µg/ml DNaseI for 90 min. The digested microvessels were added to 5 ml medium, pelleted at 500*g* for 5 min, resuspended in 500 µl of medium and divided between five wells of a 96-well filter plate. LR-BSL8.4a cells (3 × 10^4^ cells in 100 µl) were added to each well before the plate was incubated overnight, then stained with X-gal as described earlier.

### Scanning electron microscopy

Brain microvessels pre-incubated (or not) with LR-BSL8.4a cells were allowed to settle on a poly-lysine pretreated glass coverslip for 15 min, fixed in 2.5% glutaraldehyde 0.1 M phosphate buffer (pH 7.4) for 1 h and washed two times in PBS, all at room temperature. After post-fixation with 1% osmium tetroxide (Ted Pella) at for 1 h, cells were washed in deionized water, dehydrated with a graded series of ethanol immersions starting at 25–100% and critical point dried (CPD 030, Leica). The glass coverslip was then laid on an adhesive film on an SEM sample holder and firmly touched with an adhesive sample holder. The surface on which cells were deposited and the adhesive surfaces were coated with 5 nm of gold by sputter coating in a high-vacuum sputtering device (SCD005 sputter coater, Leica). The coated samples were examined with a field emission scanning electron microscope (JSM-6701F, JEOL, Japan) at an acceleration voltage of 8 kV using the in-lens secondary electron detector.

### Drug treatment

Mice infected with PbA or its luciferase-expressing derivative were injected i.p. with 0.8 mg chloroquine diphosphate (Sigma–Aldrich) 6 and 7 days p.i. and with 0.1 mg artesunate (Holly Pharmaceuticals) 6 and 6.5 days p.i. The artesunate was first dissolved in 5% sodium bicarbonate solution, and then diluted with an equal volume of saline. *Ex vivo* imaging of luciferase-expressing parasites in perfused brains and live *in vivo* imaging of the head (Supporting Information Fig 3) were performed as described previously (Claser et al, 2011).

### Folic acid challenge

To test the integrity of the blood–brain barrier, mice were injected i.v. twice with 5 mg folic acid (25 mg/ml in PBS and adjusted to neutral pH with NaOH) 1 h apart and monitored for 90 min. Mice either convulsed and died within this time or survived until they were euthanized the next day.

### Statistical analysis

Tetramer staining results were analyzed non-parametrically, using the Mann–Whitney test for two groups and the Kruskal–Wallis test with Dunn's post-test for multiple group comparison. For the brain microvessel cross-presentation experiments, the total number of blue spots arising from each brain was log-transformed allowing normal distribution of the data, followed by analysis using the two-tailed *t*-test for two groups and ANOVA with Bonferroni's post-test for multiple groups. Survival and death after folic acid challenge was analyzed with Fisher's exact test. All calculations were performed in GraphPad Prism.

## Supplementary material

As a service to our authors and readers, this journal provides supporting information supplied by the authors. Such materials are peer reviewed and may be re-organized for online delivery, but are not copy-edited or typeset. Technical support issues arising from supporting information (other than missing files) should be addressed to the authors.
