# Supplementary material for: Brain microvessel cross-presentation is a hallmark of experimental cerebral malaria
Source: EMBO Mol Med. 2013 May 16;5(7):916–31. doi: 10.1002/emmm.201202273 (PMC3721469; doi:10.1002/emmm.201202273)
Supplement: Supplementary file 1 [file emmm0005-0916-SD1.pdf]

SUPPORTING INFORMATION

Brain Microvessel Cross-presentation is a Hallmark of Experimental Cerebral Malaria

Shanshan W. Howland, Chek Meng Poh, Sin Yee Gun, Carla Claser, Benoit Malleret, Nilabh Shastri, Florent Ginhoux, Gijsbert M. Grotenbreg, Laurent Rénia

Table of Contents

Supporting Information Figures 2

S.I. Figure 1: Frequencies of SQLLNAKYL-specific cells within the entire $CD8^+$ T cell population and within the $IFN\gamma^+$ GrB^+ subset.isolated from PbA-infected mice...2

S.I. Figure 2: Supporting brain microvessel cross-presentation data 3

S.I. Figure 3: Head and brain imaging of infected mice 4

S.I. Figure 4: Parasitemia and parasite biomass following drug treatment 5

S.I. Figure 5: Brain microvessel presentation of Ovalbumin CD8 peptide … 6

Supporting Information Methods 7

Cell culture 7

Single cell TCR sequencing 7

Details of cDNA library 8

Lentivector production and transduction 9

Generation of a reporter cell line for "hosting" TCR genes 9

Generation of TCR-transduced reporter cells specific for OVA 257–264 CD8 T cell epitope 10

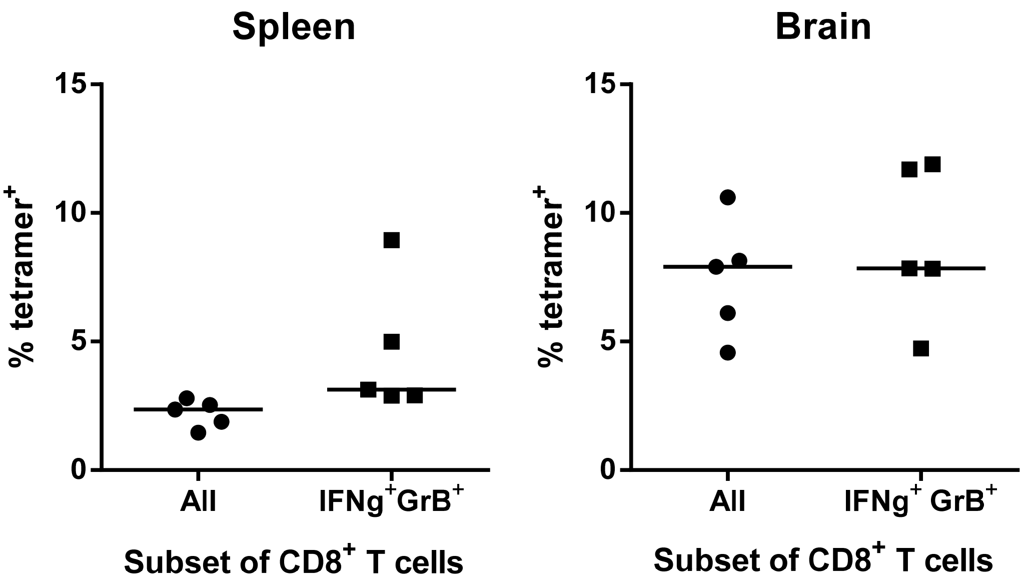

Supporting Information Figure S1. Frequencies of SQLLNAKYL-specific cells within the entire CD8$^+$ T cell population and within the IFNγ$^+$ GrB$^+$ subset isolated from PbA-infected mice. Combined tetramer and intracellular cytokine staining were performed on splenocytes (A) and brain-sequestered leukocytes (B) from PbA-infected mice 7 days p.i. The samples and gates are the same as those used in Fig. 4A, B. Bars represent medians.

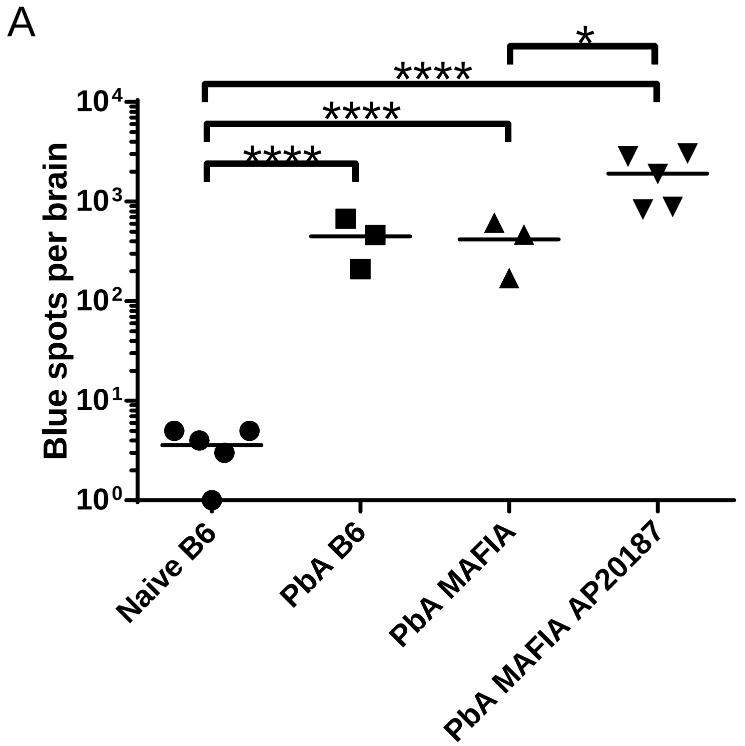

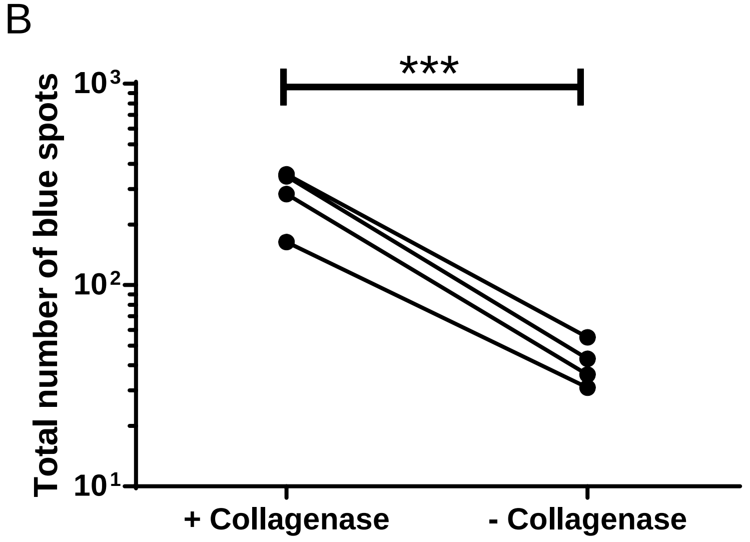

Supporting Information Figure S2. Supporting brain microvessel cross-presentation data. (A) Brain microvessel cross-presentation results from naïve C57BL/6 (B6) mice and B6 or MAFIA mice 7 days p.i. with PbA. One group of MAFIA mice was treated daily with the dimerizer drug AP20187 between 5 and 7 days p.i. Bars represent means. *** $p < 0.001$, ANOVA with Bonferroni's post test on log-transformed numbers. Data are pooled from two experiments. (B) Brain microvessels were isolated from mice 7 days post-infection with PbA, and each sample was divided into two halves. One half was digested with collagenase and DNaseI prior to co-incubation with LR-BSL8.4a cells, while collagenase was omitted for the other half. The number of blue spots resulting from each half sample was quantified after β-galactosidase staining. *** $p < 0.001$, paired t-test on log-transformed numbers.

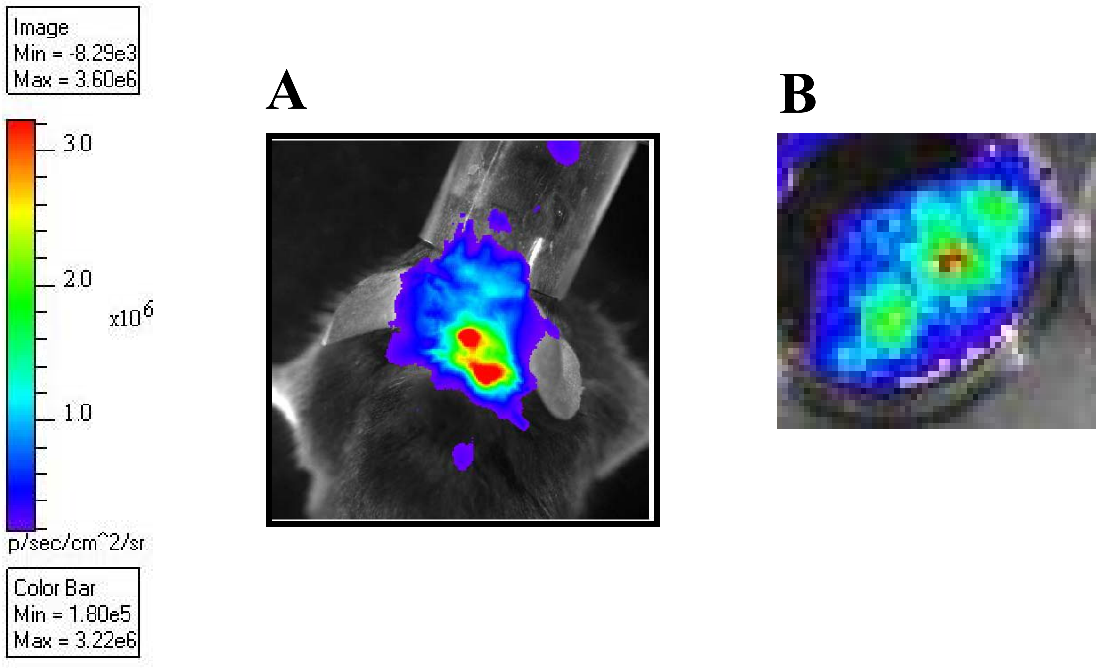

Supporting Information Figure S3. Bioimaging parameters. (A) Pseudocolor image of a mouse injected with luciferin and placed in a dorsal position for head imaging. Bioluminescence imaging was acquired with 21.7 and 4 cm FOV for whole body and head respectively, medium binning factor, and exposure time of 5-60s (changed according to the intensity of the bioluminescence signal). **(B)** Pseudocolor image of an isolated brain from a perfused animal. Brains were removed 3 min after a second subcutaneous injection of luciferin. Organs were placed in 24 well plates and imaged with 10 cm FOV, medium binning factor, and exposure times of 10-60s. To allow comparisons between images from different days, uninfected mice injected with luciferin were imaged to for background subtraction.

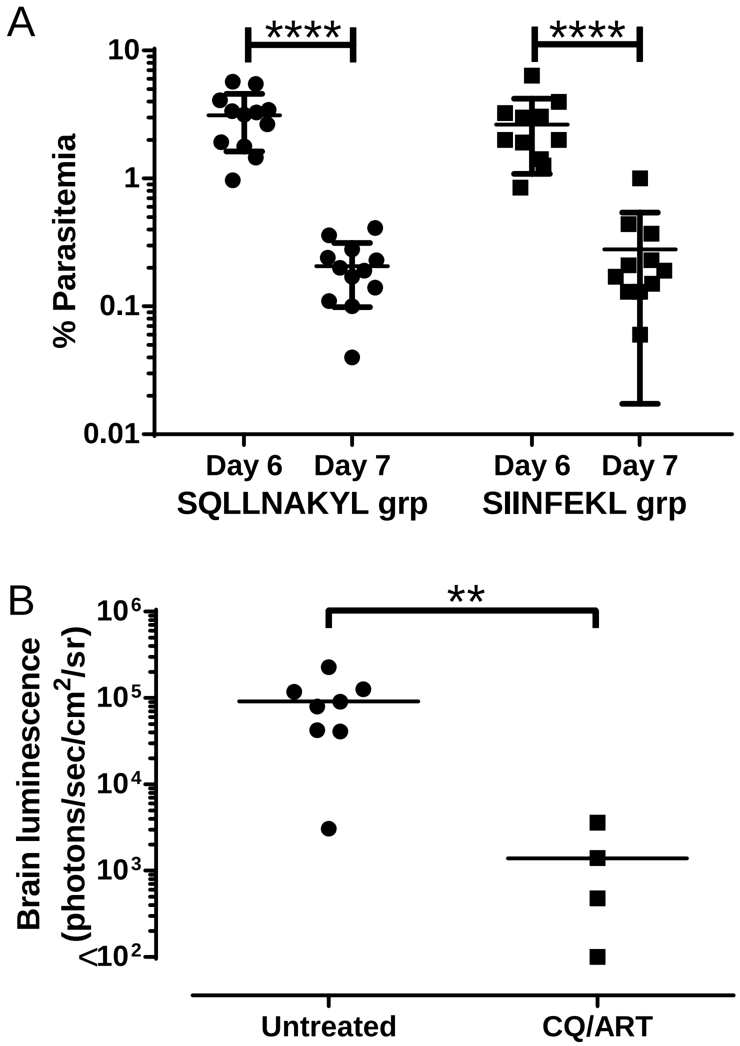

Supporting Information Figure S4. Parasitemia and parasite biomass following drug treatment. (A) Parasitemia levels of the mice in Fig. 7D were measured 6 and 7 days p.i. Bars represent mean and S.D. **** $p < 0.0001$, ANOVA with Bonferroni's post test on log-transformed values. (B) In a separate experiment, mice infected with a luciferase-transgenic derivative of PbA were either not treated (n = 8) or treated with chloroquine and artesunate (n = 4) between 6 and 7 days p.i. Two hours later, the mice were perfused and the brains were imaged for bioluminescence quantification. Bars represent the mean. ** $p < 0.01$, t test on values transformed with x' = log(x+1).

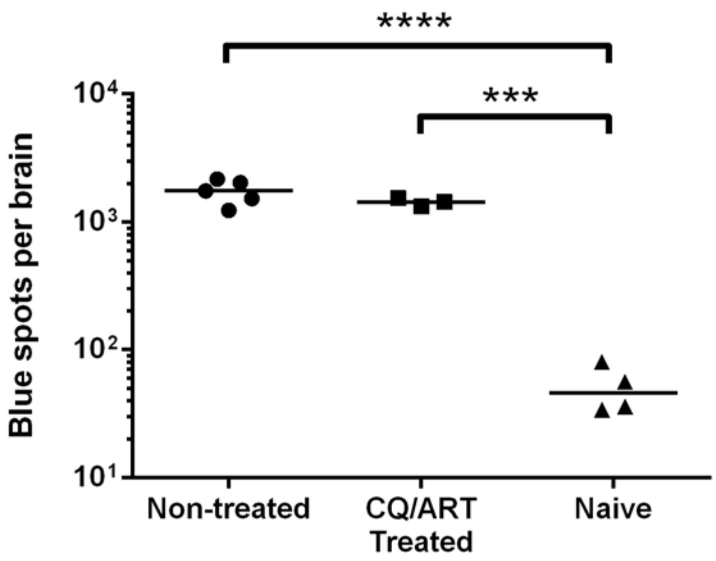

Supporting Information Figure S5. Brain microvessel presentation of ovalbumin CD8 epitope peptide. Brain microvessels were obtained from naïve C57BL/6J mice (n = 4) or mice infected with PbA that were either not treated (n = 5) or treated with chloroquine and artesunate (n = 3) between 6 and 7 days p.i. They were pulsed for 1 hour with 10^{-5} μg/ml of SIINFEKL peptide (OVA 257–264 CD8 T cell epitope) in 1 ml at 37°C, washed and incubated overnight with a reporter cell line expressing the OT-1 TCR. β-galactosidase staining was performed the next day. Bars represent means. *** $p < 0.001$, **** $p < 0.0001$, ANOVA with Bonferroni's post test on log-transformed numbers of spots.

Extended Experimental Procedures

Cell culture

The BWZ.36/CD8α cells bearing an NFAT-*lacZ* cassette were used (Sanderson and Shastri, 1994). These cells, EL4 cells (ATCC) and their derivatives were cultured in RPMI medium supplemented with 10% fetal bovine serum, 1 μM sodium pyruvate, 50 μM β-mercaptoethanol, penicillin, streptomycin and Primocin (Invivogen).

Single cell TCR sequencing

Single T cells were sorted into PCR tubes containing 2 μl of lysis/RT buffer, frozen and thawed, and then incubated at 55 °C for 1 h after adding 40 U of Superscript III (Life Technologies). The final reagent concentrations in the 2.5 μl reaction are: 1x First-Strand buffer, 10 mM DTT, 0.5 mM dNTPs, 0.8 U/μl RNaseOUT, 0.25% Nonidet P-40 (Roche), 0.1 mg/ml bovine serum albumin (NEB) and 0.1 uM each of the primers mTCRaRT and mTCRbRT. The reverse transcription product was diluted to 10 μl for tailing with oligo-dG, by adding 10 mM $MgCl_2$, 10 mM Tris-HCl pH7.5, 1 mM DTT, 2 mM dGTP, 0.75 U/ml TdT enzyme (Life Technologies) and water. The reaction was incubated at 37°C for 1 h, heat-inactivated at 70°C for 10 min, and then diluted to make up a 50 μl PCR reaction with 1 U of Phusion Hot Start II (Finnzymes), 1x GC buffer, 0.2 mM dNTPs and 0.5 μM oligo-dC-adaptor. To synthesize the second strand, the reaction was subjected to 24 cycles of amplification with annealing at 60 °C and a 90 s extension time. Each TCR gene fragment could then be amplified separately by nested PCR using Phusion in GC buffer. The first PCR contained 2 ul of cDNA in a 20 ul reaction with the primers AdaptorP1 and either mTCRa1st (for TCRα) or mTCRb1st (for TCRβ). The second PCR used 0.4 ul of the first PCR product in a 20 ul reaction with the primers AdaptorP2 and either mTCRaNest (for TCRα) or mTCRbNest (for TCRβ). Alternatively, as the sorted T cells were

Vβ8$^+$, the TCRβ junction could be directly amplified prior to 5' RACE by semi-nested PCR using CPI-Vbeta8 and mTCRb1st for the first round and CPI-Vbeta8 and mTCRbNest for the second round. As the PCR products rarely contained a single band, they were electrophoresed on a gel and the bands running around 0.5 kb were gel-extracted and cloned by TOPO TA cloning (Life Technologies) prior to DNA sequencing.

Primer name	Sequence
mTCRaRT	GTTTTCGGCACATTGATTTGG
mTCRa1st	CGGTGAACAGGCAGAGGGTG
mTCRaNest	GCAGGTTCTGGGTTCTGGATG
mTCRbRT	CTTGCCATTCACCCACCAG
mTCRb1st	CCAAGCACACGAGGGTAGCC
mTCRbNest	CCTTGGGTGGAGTCACATTTCT
oligo-dC-adaptor	ACAGCAGGTCAGTCAAGCAGTAGCAGCAGTTCGATAAGCGGCCGCCATGGACCCCCCCC CCCCDN
AdaptorP1	ACAGCAGGTCAGTCAAGCAGTA
AdaptorP2	AGCAGTAGCAGCAGTTCGATAA
CPI-Vbeta8	GAGGCTGCAGTCACCCAAA

Details of cDNA library

The upstream and downstream homologous sequences that were added to the *P. berghei* cDNA fragments to facilitate In-Fusion cloning contained a Shine-Dalgarno sequence and start codon (upstream) and stop codons (bold) in all three reading frames (downstream; **TAA**A**TAG**A**TGA**). We created a plasmid, pScrAn_Int, that when digested with NsiI and SwaI, would have ends compatible with the cDNA for In-Fusion cloning. Modified from pGEX-2T (GE Healthcare), pScrAN_Int further includes a kanamycin resistance gene whose start codon (underlined) overlaps with the third stop codon in the downstream homologous sequence. After In-Fusion cloning, the electroporated bacteria were spread on agar plates containing 50 µg/ml kanamycin and 0.02 mM IPTG, thus enriching for in-frame inserts where translational coupling enables the resistance gene to be translated.

The plasmid pool prepared from the pScrAn_Int library was then digested with EagI to release the inserts (including start and stop codons). A lentiviral transfer plasmid, pScrAn_Acc, was designed such that when digested with BstBI and AsiSI, its ends would be homologous to the inserts' ends to permit In-Fusion cloning downstream of GFP and a 2A peptide sequence (EGRGSLLTCGDVEENPGP). The In-Fusion reaction was transformed into Stbl3 bacteria (Life Technologies) and plated onto ampicillin-containing plates. To minimize the loss of library diversity, we ensured that the number of colonies obtained was at least five times that of the original library size. Plasmid purified from the scraped colonies was subsequently used to produce lentiviral particles.

Lentivector production and transduction

The transfer plasmid (1 µg for a 60 mm dish) was co-transfected with the packaging and envelope plasmids (0.35 µg pMD2.G and 0.65 µg psPAX2, kindly provided by Dr Didier Trono) into HEK 293T cells (ATCC) using EndoFectin Lenti (GeneCopoeia). The medium (DMEM + 10% FBS) was changed 8-16 h later and the lentiviral particles in the supernatant were collected after a further 48-64 h. Cells were transduced by adding variable amounts of lentiviral supernatant and 4 µg/ml Polybrene, then centrifuging at 1200 x g for 1 h at room temperature. The medium was changed after 8-16 h in the cell culture incubator.

Generation of a reporter cell line for "hosting" TCR genes

BWZ.36/CD8α cells bearing an NFAT-*lacZ* cassette were our starting point for generating reporter cell lines. However, these cells did not express *lacZ* in response to CD3 cross-linking after TCR genes and the missing CD3δ and ζ chains were co-transduced. Since other TCR signaling genes were apparently missing, hybridomas made by fusing $CD8^+$ T cells with BWZ.36/CD8α cells were passaged, sorted for loss of TCR expression, and cloned. One clone,

renamed LR-Ø, that had lost its functionally rearranged TCRβ gene and had very low level expression of its TCRα gene, was found to support *lacZ* reporting after exogenous TCR gene introduction.

Generation of TCR-transduced reporter cells specific for OVA 257–264 CD8 T cell epitope

Variable regions of the transgenic TCR specific for OVA 257–264 CD8 T cell epitope (SIINFEKL; Hogquist *et al.*, 1994) were amplified by RT-PCR from OT-1 mice (obtained from Jackson, Bar Harbor) The variable regions were assembled by PCR with the constant regions into a single open reading frame, with the two chains separated by a 2A self-cleaving peptide. TCR-expressing cells were sorted after lentivector transduction of these TCR genes into LR-Ø cells bearing an NFAT-*lacZ* cassette (see above).

References

Hogquist KA, Jameson SC, Heath WR, Howard JL, Bevan MJ, Carbone FR. 1994. T cell receptor antagonist peptides induce positive selection. Cell 76(1):17-27.
