# Supplementary material for: Brain microvessel cross-presentation is a hallmark of experimental cerebral malaria
Source: EMBO Mol Med. 2013 May 16;5(7):916–31. doi: 10.1002/emmm.201202273 (PMC3721469; doi:10.1002/emmm.201202273)
Supplement: Supplementary file 2 [file emmm0005-0916-SD2.pdf]

EMBO
Molecular Medicine

Brain Microvessel Cross-presentation is a Hallmark of Experimental Cerebral Malaria

Shanshan W. Howland, Chek Meng Poh, Sin Yee Gun, Carla Claser, Benoit Malleret, Nilabh Shastri, Florent Ginhoux, Gijsbert M. Grotenbreg, Laurent Rénia

Corresponding author: Laurent Rénia, Singapore Immunology Network

Review timeline:

Submission date:	14 November 2012
Editorial Decision:	03 January 2013
Revision received:	17 March 2013
Editorial Decision:	04 April 2013
Revision received:	04 April 2013
Accepted:	05 April 2013

Transaction Report:

(Note: With the exception of the correction of typographical or spelling errors that could be a source of ambiguity, letters and reports are not edited. The original formatting of letters and referee reports may not be reflected in this compilation.)

Editor: Natascha Bushati / Céline Carret

1st Editorial Decision 03 January 2013

Thank you for the submission of your manuscript to EMBO Molecular Medicine. We have now heard back from the three referees whom we asked to evaluate your manuscript.

You will see that while all three reviewers are generally supportive of your work and underline its considerable potential interest, they also raise a number of specific concerns that prevent us from considering publication at this time.

Importantly, all three reviewers highlight that more evidence for the role of brain microvascular endothelial cells in antigen presentation is required and suggest analysing the purity of the microvessel preparations in both wild-type and MAFIA mice. In addition, reviewer 1 suggests to perfuse the brains upon infection with different parasites, to determine whether CD8 T cells accumulate differentially in the brain. Reviewer 2 suggests that direct evidence of cerebral malaria, such as brain pathology and Evans blue test, should be provided in the experiment presenting the treatment approach.

Finally, all three reviewers, including reviewer 3, have a number of important suggestions to improve interpretation of your data, impact and readability of your work.

Should you be able to address the raised concerns with additional experiments where appropriate, we would be willing to consider a revised manuscript.

Please note that it is EMBO Molecular Medicine policy to allow a single round of revision in order to avoid the delayed publication of research findings. Consequently, acceptance or rejection of the

© EMBO

manuscript will depend on the completeness of your responses included in the next version of the manuscript.

EMBO Molecular Medicine has a "scooping protection" policy, whereby similar findings that are published by others during review or revision are not a criterion for rejection. Should you decide to submit a revised version, I do ask that you get in touch after three months if you have not completed it, to update us on the status.

Please also contact us as soon as possible if similar work is published elsewhere. If other work is published we may not be able to extend the revision period beyond three months.

I look forward to receiving your revised manuscript.

***** Reviewer's comments *****

Referee #1 (Comments on Novelty/Model System):

Technical quality is clear. Novelty: no one has shown presentation of malaria antigens from brain blood vessels during experimental cerebral malaria. Medical impact is medium as it is not clear that CD8 T cells mediate disease in human malaria. This is a highly controversial issue and divides the field, but in my opinion it is yet to be resolved either way. Hence, the adequacy of the is "unclear at this stage". This means that the findings may eventually be highly relevant or potentially irrelevant, depending on whether the mouse model pans out to be reflective of the human model.

Referee #1 (General Remarks):

The report by Howland et al provides some very interesting findings that extend our understanding of the immunopathology associated with experimental cerebral malaria. The report identifies a new blood stage MHC I-restricted antigen that is expressed by several Plasmodium species. Evidence is provided that CD8 T cells specific for this antigen migrate to the brain during infection with various species of Plasmodium, and the authors show that for P.berghei ANKA infection but not infection by other species, this antigen is cross-presented by cells of the brain microvessels. Although these authors were unable to define the precise cell type cross-presenting parasite antigen, their work nicely supports other studies implicating endothelial cells in this function. They also provide evidence that reducing parasite burden by drug treatment late in disease may be protective through its reduction in antigen cross-presentation by the cells of the brain microvessels.

Introduction: The authors state "... not one CD8 epitope has been identified...". But later point out that Lau et al have identified several epitopes. The authors should modify this statement to say "...very few epitopes..."

Figure 5 shows evidence of CD8 T cells accumulating in the brains of mice infected with non-CM causing parasites. As the authors do no perfuse the mice/brains, it is unclear whether this apparent accumulation is simply reflecting their presence in the blood, without any specific sequestration in the brain. As we might expect these cells to be in the blood after expansion in the spleen and release into the circulation, I think it is import to determine whether there is a difference in T cell sequestration in the brain after perfusion for the different infections.

Figure 6. It is not clear how many times this experiment was performed. Please indicate this throughout the manuscript so that the reader can be confident these experiments are not single experiments. While this figure shows preferential presentation of Ag by the vessel material isolated from the brains of PbA infected mice, there is minimal evidence that presentation is mediated by endothelial cells. I assume there are many cell types present in the vessel mixture. Is it possible to digest cells into single cells then sort for markers to indicate presentation by endothelial cells? The MAFIA mouse experiment requires side-by-side comparison to normal infected controls to demonstrate there is no reduction in presentation.

Figure 3. "(G)" is missing next to "brain" in the legend to figure 3.

Referee #2 (Comments on Novelty/Model System):

Studies in Fig. 1-5 were performed in high quality both technically and scientifically. Unfortunately, since other group identified CD8 epitope expressed in blood stage of malaria parasites, this study is not the first to identify the epitope. Yet, authors were able to generate MHC tetramer and identify specific T cells, which should be highly evaluated. However, studies in Fig. 6 and 7 are not satisfactory. Information regarding the experimental conditions such as purity of microvessel preparation is lacking, and control groups are not sufficient. If these points are improved, I think that the manuscript may be qualified for publication.

Referee #2 (General Remarks):

In this paper, authors identified a major CD8 epitope that is conserved across rodent malaria parasites. Using MHC tetramer, they found that epitope-specific T cells were induced during infection with PbA and accumulate in brain. Interestingly, accumulation of these specific CD8 T cells was observed during infection with non-ECM-causing parasites. Authors showed that brain microvessels prepared from PbA-infected mice were able to cross-present the epitope, while those prepared from non-ECM-causing parasites were not. Studies on the identification of the epitope and its specific T cells are performed well. However, I have some concerns with regard to the antigen-presentation by brain microvessels.

Specifically,

1. Authors prepared brain microvessels from PbA-infected mice and showed that they can present malaria antigen in Fig. 6B. They also showed microvessels prepared from PbNK65- and Py17X-infected brain were not able to present SQLLNAKYL-epitope. However, they did not show the purity of the preparation and the proportions of contaminating hematopoietic cells. It is crucial to show that the difference in antigen presentation is not due to the composition of cell types in the microvessel preparations. Also, it is important to show the levels of MHC on microvessels and their ability of antigen-presentation when SQLLNAKYL peptide was exogenously added in culture.

2. Authors used MAFIA transgenic mice to rule out the possible contamination of antigen-presenting cells in microvessel-preparation in Fig. 6D. First, the control of the drug-treated PbA-MAFIA, untreated group, is not shown. Second, myeloid cells are not completely removed in drug-treated MAFIA transgenic mice, and it is again important to show the purity of cell population in this experiment. It is crucial to show that hematopoietic cells are not involved in antigen presentation of SQLLNAKYL-epitope during PbA-infection. Anti-CD45 antibody may help to rule out the involvement of hepatopoietic cells. For example, one can treat microvessel preparation with anti-CD45 mAb and complement to remove residual hematopoietic cells.

3. In Fig. 7C, the purity of microvessels and their MHC expression should be shown. It is possible that CG/ART-treatment reduced the number of contaminating antigen presenting cells and thus reduced the response.

4. In Fig. 7DE, the reason for the death of mice is not clearly shown. Authors should show the evidence of cerebral malaria, such as brain pathology and Evans blue test for the BBB integrity.

5. The title is misleading, if authors are not able to conclude that microvessels are responsible for the cross-presentation as described in page 15 line 16-8.

Referee #3 (General Remarks):

This ms reports by Howland and coauthors reports on a timely and interesting subject. The authors report on three important discoveries: first, they identify a natural target of the CTL response against plasmodial antigens during experimental infection; second, they reveal a role for cross-presentation of this plasmodial antigen in the pathogenesis of cerebral malaria and third, they reveal an important difference of distinct strains of plasmodia for being cross-presented locally in the brain. The study is very elegant, carefully conducted and adequately controlled.

A few issues require attention:
- Isolation of brain microvessels for ex vivo cross-presentation assays is not controlled for purity of the brain microvascular endothelial cells. The experiments shown here do not adequately identify brain microvascular endothelial cells as cross-presenting cells. Peptide injection will lead to antigen-presentation by all MHC-I expressing cells, not specifically by brain endothelial cells. In Mafia mice, depletion of macrophages, monocytes, dendritic cells is incomplete making it impossible to discriminate between endothelial cells or myeloid cells acting as cross-presenting cells. The authors should either provide more convincing data for endothelial cell cross-presentation or phrase more carefully the title, results and discussion. This is particular relevant with respect to the mechanism of endothelial damage inflicted by CTLs activated by cross-presentation, which may either result from direct cell death induced by MHC-I recognition on the endothelial cell or by a recently described non-canonical CTL effector function that operates via TNF (Wohlleber et al, Cell Reports 2012).
- It remains unclear why there is no local cross-presentation in the brain of the other plasmodium strains that do not cause cerebral malaria as they also express the antigen recognized by the CTLs. The authors should discuss this issue or provide more experimental data.
- The rapid loss of cross-presentation following drug treatment and the resultant drop in parasitemia suggests rapid turnover of peptide loaded MHC I molecules from the surface of the cross-presenting cell. As this is also clinically potentially relevant, the authors should at least discuss the current knowledge in the literature on the dynamics of cross-presentation in myeloid cells and endothelial cells.

1st Revision - authors' response 17 March 2013

Editor's comments

You will see that while all three reviewers are generally supportive of your work and underline its considerable potential interest, they also raise a number of specific concerns that prevent us from considering publication at this time.

We are happy that the reviewers found values in the work presented.

Importantly, all three reviewers highlight that more evidence for the role of brain microvascular endothelial cells in antigen presentation is required and suggest analysing the purity of the microvessel preparations in both wild-type and MAFIA mice. In addition, reviewer 1 suggests to perfuse the brains upon infection with different parasites, to determine whether CD8 T cells accumulate differentially in the brain. Reviewer 2 suggests that direct evidence of cerebral malaria, such as brain pathology and Evans blue test, should be provided in the experiment presenting the treatment approach.

All these questions have been answered (see reviewers' answers).

Response to all three reviewer's concerns regarding microvessel purity and contamination by hematopoietic/myeloid cells:

Even after collagenase digestion, the microvessel preparations consist of multicellular tubes, composed of endothelial cells, pericytes and microglial cells, and possibly brain-sequestered

leukocytes. We have made this clearer in the results and discussion text as well as by providing SEM images of the vessels. Extended digestion to obtain single cells greatly decreases viability, in our hands and as reported by Song and Pachter (In Vitro Cell Dev Biol Anim, 2003, 39: 313-320). We are therefore unable to analyse the cell type composition of the microvessels by flow cytometry.

Instead of analysing the purity of the preparations, we have taken the opposite approach of quantifying (in the new experiment presented in the revised manuscript) the maximum possible contribution of hematopoietic/myeloid cells to cross-presentation.

When we intentionally purified brain-sequestered leukocytes (including CD45+ CD11b+ Gr1+ monocytes, macrophages, and granulocytes) from PbA-infected mice and incubated them with LR-BSL8.4a cells in the same way that the brain microvessel cross-presentation assay is performed, we saw that leukocytes from PbA-infected mice did produce a cross-presentation signal, but less than one-tenth of the signal resulting from microvessel preparations (Fig. 6C). Therefore, even in the worst case scenario where no myeloid cells are removed during microvessel purification, ~90% of the microvessel cross-presentation signal must come from the microvessels per se.

We have also repeated the MAFIA mouse experiment with the additional controls of infected C57BL/6 mice (requested by reviewer 1) and infected MAFIA mice not treated with dimerizer (requested by reviewer 2). There was no reduction (and even an apparent increase) in brain microvessel cross-presentation by dimerizer-treated MAFIA mice compared to either of these controls (SI Fig 3A). Even though depletion is not fully complete (around 80%) in treated MAFIA mice, the data argues against myeloid cells being responsible for the detected cross-presentation.

Please note that we do not claim that endothelial cells are necessarily the cross presenting cells; in particular, the title and abstract refer only to brain microvessels. Nevertheless, we have provided two more lines of evidence to support endothelial cells being the cross-presenting population (although we cannot rule out pericytes).

First, omitting collagenase from the microvessel isolation process decreases the detected cross-presentation by 80-90% (SI Fig 2B). The purpose of collagenase digestion is not to break the vessels into smaller fragments but to erode the vessel basal lamina (Song and Pachter, In Vitro Cell Dev Biol Anim, 2003, 39: 313-320). Since the basal lamina would largely prevent the reporter cells from coming into contact with endothelial cells and pericytes, these cell types are more likely to be the cross-presenting cells, as opposed to astrocytes or microglia that are already exposed.

Second, we have provided SEM images (Fig 6E-F) of reporter cells forming cell-cell junctions with microvessel walls (both the ablumenal and lumenal surfaces). These junctions are likely to be immunological synapses with the cross-presenting cells, which appear to be endothelial cells (although again, pericytes cannot be ruled out).

Finally, all three reviewers, including reviewer 3, have a number of important suggestions to improve interpretation of your data, impact and readability of your work.

Their suggestions have been taken into consideration and we have modified the manuscript accordingly.

Reviewer's comments

Referee #1 (Comments on Novelty/Model System):

Technical quality is clear. Novelty: no one has shown presentation of malaria antigens from brain blood vessels during experimental cerebral malaria. Medical impact is medium as it is not clear that CD8 T cells mediate disease in human malaria. This is a highly controversial issue and divides the field, but in my opinion it is yet to be resolved either way. Hence, the adequacy of this is "unclear at this stage". This means that the findings may eventually be

highly relevant or potentially irrelevant, depending on whether the mouse model pans out to be reflective of the human model.

We thank the reviewer for his supportive and unbiased comments.

Referee #1 (General Remarks):

The report by Howland et al provides some very interesting findings that extend our understanding of the immunopathology associated with experimental cerebral malaria. The report identifies a new blood stage MHC I-restricted antigen that is expressed by several Plasmodium species. Evidence is provided that CD8 T cells specific for this antigen migrate to the brain during infection with various species of Plasmodium, and the authors show that for P. berghei ANKA infection but not infection by other species, this antigen is cross-presented by cells of the brain microvessels. Although these authors were unable to define the precise cell type cross-presenting parasite antigen, their work nicely supports other studies implicating endothelial cells in this function. They also provide evidence that reducing parasite burden by drug treatment late in disease may be protective through its reduction in antigen cross-presentation by the cells of the brain microvessels.

We thank the reviewer for his supportive comments.

1. Introduction: The authors state "... not one CD8 epitope has been identified...". But later point out that Lau et al have identified several epitopes. The authors should modify this statement to say "...very few epitopes..."

The statement was meant to explain the motivation/situation when we began the study and has been modified to be more explicit: "... not one CD8 epitope had yet been identified in C57BL/6 mice at the start of this work ...".

2. Figure 5 shows evidence of CD8 T cells accumulating in the brains of mice infected with non-CM causing parasites. As the authors do no perfuse the mice/brains, it is unclear whether this apparent accumulation is simply reflecting their presence in the blood, without any specific sequestration in the brain. As we might expect these cells to be in the blood after expansion in the spleen and release into the circulation, I think it is import to determine whether there is a difference in T cell sequestration in the brain after perfusion for the different infections.

Although the mice were not perfused, we did bleed the mice retro-orbitally (under anaesthesia) until they were dead, thus almost all of the circulating blood is removed from the brains. The remaining CD8 T cells left in the brain are largely specifically sequestered, as is apparent from the proportion of IFNg+GrB+ CD8 T cells in the brain (~80%) versus the spleen (~30%) (Fig 4A). We did perform a comparative analysis of exsanguinated and perfused mice. We did not see a significant difference in either the total number of CD8 T cells in the brain or the number of tetramer-positive CD8 T cells in the brain. This last point has been added to the Materials and Methods.

3. Figure 6. It is not clear how many times this experiment was performed. Please indicate this throughout the manuscript so that the reader can be confident these experiments are not single experiments.

Fig 6B is representative of 3 experiments. Figure legends have been annotated when experiments have been repeated.

4. While this figure shows preferential presentation of Ag by the vessel material isolated from the brains of PbA infected mice, there is minimal evidence that presentation is mediated by endothelial cells.

Please see relevant response above directed to all three reviewers.

5. I assume there are many cell types present in the vessel mixture. Is it possible to digest cells into single cells then sort for markers to indicate presentation by endothelial cells?

No, the cells die when digested to this extent.

6. The MAFIA mouse experiment requires side-by-side comparison to normal infected controls to demonstrate there is no reduction in presentation.

We have repeated the experiment with infected B6 mice (SI Fig 3A) and there is indeed no reduction. These data have been added and described to the revised manuscript.

7. Figure 3. "(G)" is missing next to "brain" in the legend to figure 3.

Fixed.

Referee #2 (Comments on Novelty/Model System):

1, Studies in Fig. 1-5 were performed in high quality both technically and scientifically. Unfortunately, since other group identified CD8 epitope expressed in blood stage of malaria parasites, this study is not the first to identify the epitope. Yet, authors were able to generate MHC tetramer and identify specific T cells, which should be highly evaluated. However, studies in Fig. 6 and 7 are not satisfactory. Information regarding the experimental conditions such as purity of microvessel preparation is lacking, and control groups are not sufficient. If these points are improved, I think that the manuscript may be qualified for publication.

We thank the reviewer for his comments. We have added new experiments and experimental information for Figure 6 and 7.

Referee #2 (General Remarks):

In this paper, authors identified a major CD8 epitope that is conserved across rodent malaria parasites. Using MHC tetramer, they found that epitope-specific T cells were induced during infection with PbA and accumulate in brain. Interestingly, accumulation of these specific CD8 T cells was observed during infection with non-ECM-causing parasites. Authors showed that brain microvessels prepared from PbA-infected mice were able to cross present the epitope, while those prepared from non-ECM-causing parasites were not. Studies on the identification of the epitope and its specific T cells are performed well. However, I have some concerns with regard to the antigen-presentation by brain microvessels.

Specifically,

1. Authors prepared brain microvessels from PbA-infected mice and showed that they can present malaria antigen in Fig. 6B. They also showed microvessels prepared from PbNK65- and Py17X-infected brain were not able to present SQLLNAKYL-epitope. However, they did not show the purity of the preparation and the proportions of contaminating hematopoietic cells. It is crucial to show that the difference in antigen presentation is not due to the composition of cell types in the microvessel preparations.

Please see relevant response above directed to all three reviewers.

Also, it is important to show the levels of MHC on microvessels and their ability of antigen-presentation when SQLLNAKYL peptide was exogenously added in culture.

As suggested by the reviewer, we did try to show levels of MHC Class I on isolated microvessels by immunofluorescence microscopy. However, we were not able to obtain good

and reproducible staining. We believe it is due to the collagenase treatment to obtain the microvessels. It has to be noted that our presentation assay takes 24 h, a time that may be necessary for surface re-expression of MHC loaded with parasite-derived peptides. To support this hypothesis, we performed the cross-presentation assay 1 or 3 hours post-purification and did not observe any response.

Nevertheless, we performed the *in vitro* assay with the addition of the peptide SIINFEKL corresponding to the CD8 epitope of ovalbumin and the use of a reporter cell line recognizing the OVA peptide in the context of MHC class I molecule. We chose this peptide over the SQLLNAKYL peptide to eliminate any cofounding factor due to endogenous presentation. We observed that exogenous addition of the OVA peptide to the microvessels from PbA-infected mice induces higher numbers of blue spots than microvessels from naive mice. This means i) that after 24 hours, MHC class I are re-expressed at the surface of microvessel cells and ii) that more endogenous MHC molecules were re-expressed for microvessel cells from PbA-infected mice than those of naive mice and iii) that drug treatment had no effect on the level of MCH re-expression. These data have been added to and discussed in the revised manuscript as SI Fig 5.

2. Authors used MAFIA transgenic mice to rule out the possible contamination of antigen-presenting cells in microvessel-preparation in Fig. 6D. First, the control of the drug-treated PbA-MAFIA, untreated group, is not shown.

We have repeated the experiment with infected MAFIA mice not treated with the dimerizer drug (SI Fig 2A). The treated MAFIA mice did not show a reduction in cross-presentation compared to the untreated MAFIA mice; on the contrary, there was a significant increase. This may possibly indicate that certain myeloid subsets may either be competing for parasite antigen or play a role in down-regulating cross-presentation. These data have been added and described to the revised manuscript.

3. Second, myeloid cells are not completely removed in drug-treated MAFIA transgenic mice, and it is again important to show the purity of cell population in this experiment.

Incomplete depletion of myeloid cells is unlikely to account for the cross-presentation seen with treated MAFIA mice since there was no reduction at all with ~80% depletion. We have addressed the purity concern by showing that the cross-presentation contribution of all brain-sequestered leukocytes is but a small fraction of cross-presentation by the microvessel preparation (Fig 6C). These data have been added and described to the revised manuscript.

4. It is crucial to show that hematopoietic cells are not involved in antigen presentation of SQLLNAKYL-epitope during PbA-infection. Anti-CD45 antibody may help to rule out the involvement of hematopoietic cells. For example, one can treat microvessel preparation with anti-CD45 mAb and complement to remove residual hematopoietic cells.

As described above, we have addressed the role of hematopoietic cells by intentionally purifying them and quantifying their contribution to the cross-presentation signal. We thank the reviewer for the interesting suggestion of anti-mouse CD45 and complement but conditions need to be optimized, which is difficult to do without a good way to analyse the purity of the microvessels in the first place.

5. In Fig. 7C, the purity of microvessels and their MHC expression should be shown. It is possible that CG/ART-treatment reduced the number of contaminating antigen presenting cells and thus reduced the response.

See above response to reviewer I. Further, in the new experiment where exogenous SIINFEKL peptide was added to microvessels to measure MHC class I expression (SI Fig

5A), microvessels from drug-treated mice induced the same response as non-treated mice. Thus drug treatment had no effect on MHC class I expression.

6. In Fig. 7DE, the reason for the death of mice is not clearly shown. Authors should show the evidence of cerebral malaria, such as brain pathology and Evans blue test for the BBB integrity.

As described in the Results, the mice in these experiments died when the neurotoxin folate crossed the BBB, causing convulsions. Folate has a direct epileptogenic effect on neurons, causing convulsions and death if it can access the central nervous system via a breach in the blood-brain barrier (Hommes et al., 1973, J Neur Sci, 19, 63; Remler and Marcussen, Epilepsia, 1984, 25, 574). At the concentration used, folate does not induce mortality in mice with an intact BBB.

The folic acid assay thus tests only this one aspect of ECM pathology i.e. BBB integrity. Since the BBB damage was inflicted only by $CD8^+$ T cells of one specificity, it is reasonable that we observed only mild/early signs of ECM. We did not perform brain histology because we expect to see much less pathology compared to "normal" ECM caused by many more parasite-specific $CD8^+$ T cells. Before turning to the folic acid challenge, we did perform a pilot experiment with the Evans blue test and saw a significant difference ($p = 0.011$) between SQLLNAKYL-injected and control peptide-injected brains *extracted* with DMF (see below). However, the experiment group brains were only light blue (not dark blue like full ECM) and thorough perfusion was needed for the differences to be significant, which is why we switched to the more sensitive folic acid assay.

We have commented on the limitations of the folic acid assay in the discussion by adding the following: One limitation of our approach is that blood-brain barrier integrity was assessed by this very sensitive assay, by injecting folic acid intravenously. Folate has a direct epileptogenic effect on neurons, causing convulsions and death if it can access the central nervous system via a breach in the blood-brain barrier (Hommes et al., 1973).Without performing the folic acid assay, the damage caused by $CD8^+$ T cells of just this one specificity was insufficient to recapitulate the full extent and range of pathologies of ECM. It has to be reminded that SQLLNAKYL-specific CD8 T cells represent at best 10-15% of the total brain-sequestered CD8 T cells and thus other specificities likely participate in the ECM pathologies.

7. The title is misleading, if authors are not able to conclude that microvessels are responsible for the cross-presentation as described in page 15 lines 16-8.

As described above, we have added 3 lines of evidence to support the claim that microvessels are indeed responsible for the cross-presentation.

Referee #3 (General Remarks):

This ms reports by Howland and co-authors reports on a timely and interesting subject. The authors report on three important discoveries: first, they identify a natural target of the CTL response against plasmodial antigens during experimental infection; second, they reveal a role for cross-presentation of this plasmodial antigen in the pathogenesis of cerebral malaria and third, they reveal an important difference of distinct strains of plasmodia for being cross-presented locally in the brain. The study is very elegant, carefully conducted and adequately controlled.

A few issues require attention:

1. Isolation of brain microvessels for ex vivo cross-presentation assays is not controlled for purity of the brain microvascular endothelial cells. The experiments shown here do not adequately identify brain microvascular endothelial cells as cross-presenting cells. Peptide injection will lead to antigen-presentation by all MHC-I expressing cells, not specifically by brain endothelial cells. In Mafia mice, depletion of macrophages, monocytes, dendritic cells is incomplete making it impossible to discriminate between endothelial cells or myeloid cells acting as cross-presenting cells. The authors should either provide more convincing data for endothelial cell cross-presentation or phrase more carefully the title, results and discussion.

Please see relevant response above (editor response) directed to all three reviewers.

2. This is particular relevant with respect to the mechanism of endothelial damage inflicted by CTLs activated by cross-presentation, which may either result from direct cell death induced by MHC-I recognition on the endothelial cell or by a recently described non-canonical CTL effector function that operates via TNF (Wohlleber et al, Cell Reports 2012).

The work by Wohlleber et al. is very exciting, but this non-canonical effector function is unlikely to play a major role in ECM since TNFα knockout mice remain susceptible to ECM (Engwerda et al., JEM 2002). We have mentioned this in the discussion.

3. *It remains unclear why there is no local cross-presentation in the brain of the other plasmodium strains that do not cause cerebral malaria as they also express the antigen recognized by the CTLs. The authors should discuss this issue or provide more experimental data.*

In the previous manuscript version, we proposed that the difference in local cross-presentation between ECM and Non-ECM strains is the capacity of ECM strains to display higher sequestration in the brains of infected mice. We are now providing new data (new Figure 7) using luciferase-tagged parasite bioluminescence measurements showing that there is a difference in parasite accumulation in the brains of mice with an ECM strain (PbA) versus a non-ECM strain (*P. yoelii* YM).

4. *The rapid loss of cross-presentation following drug treatment and the resultant drop in parasitemia suggests rapid turnover of peptide loaded MHC I molecules from the surface of the cross-presenting cell. As this is also clinically potentially relevant, the authors should at least discuss the current knowledge in the literature on the dynamics of cross-presentation in myeloid cells and endothelial cells.*

We agree with the reviewer that the rapid loss of cross-presentation may suggest a rapid turn-over of peptide loaded MHC I molecules from the surface of the cross-presenting cell and this is supported by data presented on Supplemental Figure 4.

This may be due also to the fact that presentation of PbA antigens depends on the parasite load in the brain and that a certain threshold is needed to lead to ECM. Since we treated the mice the day before ECM onset, the threshold may not have been yet reached.

We have added a section in the discussion on the current knowledge on the dynamics of cross-presentation in myeloid cells and endothelial cells.

2nd Editorial Decision 04 April 2013

We have now received the enclosed reports from the referees that were asked to re-assess your manuscript. As you will see, the reviewers are now supportive and I am happy to inform you that we will be able to accept your manuscript pending editorial amendments.

I look forward to seeing a new revised version of your manuscript as soon as possible.

***** Reviewer's comments *****

Referee #1 (Comments on Novelty/Model System):

Technical quality is clear. Novelty: no one has shown presentation of malaria antigens from brain blood vessels during experimental cerebral malaria. Medical impact is medium as it is
not clear that CD8 T cells mediate disease in human malaria. This is a highly controversial issue and divides the field, but in my opinion it is yet to be resolved either way. This means that the findings may eventually be highly relevant or potentially irrelevant, depending on whether the mouse model pans out to be reflective of the human model.

Referee #1 (General Remarks):

I am satisfied with the revisions.

Referee #2 (Comments on Novelty/Model System):

As I stated in the initial review, identification of CD8 epitope in blood stage malaria parasites and studies on the specific T cells using MHC tetramer were performed in high standard and quality. It is novel that authors showed that the epitope-specific T cells are present in the brain of mice that do not develop ECM and that brain microvessels can cross-present malaria antigen to CD8 T cells in mice that develop ECM. Authors also provided sufficient background information regarding the ability of brain microvessels to present malaria antigen to CD8 T cells. While it is controversial whether CD8 T cells are involved in the etiology of human CM, it is important to determine the mechanisms underlying the pathogenesis of CM using animal models, which allow experimental approach.

Referee #2 (General Remarks):

Authors responded adequately to most of my concerns. While authors did not provide the information regarding the purity of microvessel preparation, they provided supportive information indicating that the antigen-presentation by microvessels was not due to the contaminating hematopoietic cells. The interpretation of the data was carefully performed. I think that this study is a significant advance in the specific field and provides interesting information to broad non-specialists.
